# Phosphorylation of axin within biomolecular condensates counteracts its tankyrase-mediated degradation

**DOI:** 10.1242/jcs.261214

**Published:** 2023-10-27

**Authors:** Katharina Klement, Martina Brückner, Dominic B. Bernkopf

**Affiliations:** Experimental Medicine II, Nikolaus-Fiebiger-Center, Friedrich-Alexander University Erlangen-Nürnberg, 91054 Erlangen, Germany

**Keywords:** Axin, Wnt signaling, Biomolecular condensates, Tankyrase

## Abstract

Axin (also known as AXIN1) is a central negative regulator of the proto-oncogenic Wnt/β-catenin signaling pathway, as axin condensates provide a scaffold for the assembly of a multiprotein complex degrading β-catenin. Axin, in turn, is degraded through tankyrase. Consequently, tankyrase small-molecule inhibitors block Wnt signaling by stabilizing axin, revealing potential for cancer therapy. Here, we discovered that axin is phosphorylated by casein kinase 1 alpha 1 (CSNK1A1, also known as CK1α) at an N-terminal casein kinase 1 consensus motif, and that this phosphorylation is antagonized by the catalytic subunit alpha of protein phosphatase 1 (PPP1CA, hereafter referred to as PP1). Axin condensates promoted phosphorylation by enriching CK1α over PP1. Importantly, the phosphorylation took place within the tankyrase-binding site, electrostatically and/or sterically hindering axin–tankyrase interaction, and counteracting tankyrase-mediated degradation of axin. Thus, the presented data propose a novel mechanism regulating axin stability, with implications for Wnt signaling, cancer therapy and self-organization of biomolecular condensates.

## Teaser

**Highlighted Article:** Within biomolecular condensates the Wnt pathway regulator axin is N-terminally phosphorylated by CK1α, which impairs axin–tankyrase interaction and protects axin from tankyrase-mediated degradation.

## INTRODUCTION

The Wnt/β-catenin signaling pathway controls patterning of body axes during embryonic development as well as stem cell fate in diverse adult epithelia, explaining its pivotal roles in tissue homeostasis and regeneration (Clevers and Nusse, 2012; Petersen and Reddien, 2009). Not surprisingly, deregulation of the pathway is causally associated with severe pathologies, such as cancer development, particularly in the large intestine (Clevers and Nusse, 2012). Axin (also known as AXIN1) is a central negative regulator of the Wnt/β-catenin pathway (Fagotto et al., 1999; Ikeda et al., 1998), which helps to tightly control the levels of the transcriptional co-factor β-catenin. Axin functions as a scaffold protein, recruiting adenomatous polyposis coli (APC), casein kinase 1 family proteins (CSNK1s, hereafter referred to as CK1), glycogen synthase kinase 3β (GSK3B) together with β-catenin in a multiprotein complex (Behrens et al., 1998; Ikeda et al., 1998; Stamos and Weis, 2013). This β-catenin destruction complex mediates phosphorylation of β-catenin by CK1 and GSK3B, thereby inducing its subsequent proteasomal degradation, which silences the pathway (Stamos and Weis, 2013). Binding of Wnt ligands to receptors of the Frizzled, and the low-density lipoprotein receptor-related proteins 5 and 6 (LRP5 and LRP6) families activates the pathway. The activated receptors recruit positive pathway regulators of the dishevelled family and inhibit the β-catenin destruction complex, allowing accumulation of β-catenin and transcription of its target genes (MacDonald and He, 2012). Uncontrolled pathway activation can have fatal consequences. Genetic inactivation of axin in mice, for example, results in axis duplication effects with embryonic lethality (Zeng et al., 1997), illustrating the importance of the Wnt pathway in general, as well as the importance of axin for pathway regulation.

Axin has a highly related homolog, axin2 (also known as conductin), similarly promoting β-catenin degradation (Behrens et al., 1998). In contrast to the ubiquitous expression of axin, axin2 is a β-catenin target gene regulating the pathway in a negative feedback loop (Jho et al., 2002; Leung et al., 2002; Lustig et al., 2002). Axin activity critically depends on polymerization through its C-terminal dishevelled and axin (DIX) domain, giving rise to dynamic phase-separated, biomolecular condensates, which are microscopically visible as cytosolic spheres when axin is overexpressed in various human cell lines (Fagotto et al., 1999; Fiedler et al., 2011; Kishida et al., 1999; Nong et al., 2021; Schwarz-Romond et al., 2007b). Axin condensates promote the assembly of the β-catenin destruction complex and the consequent phosphorylation of β-catenin, probably by increasing the functional affinities for the interactors through high local concentration – as discussed for antibody avidity (Fiedler et al., 2011). In addition, biomolecular condensates have been suggested to concentrate or exclude certain factors of enzymatic reactions to control reaction specificity (Banani et al., 2017), and axin condensates may enrich kinases over phosphatases to favor β-catenin phosphorylation. Axin activity is regulated through post-translational modifications. Phosphorylation of axin by CK1 increases GSK3B binding (Luo et al., 2007), while phosphorylation by GSK3B, in turn, increase β-catenin binding and axin stability (Jho et al., 1999; Willert et al., 1999; Yamamoto et al., 1999). In contrast, poly ADP-ribosylation (PARylation) of axin or axin2 by tankyrase marks axin proteins for proteasomal degradation and decreases their stability (Huang et al., 2009). Small-molecule tankyrase inhibitors block Wnt signaling by stabilizing axin and axin2 (Huang et al., 2009), and next-generation tankyrase inhibitors are currently developed for targeted colorectal cancer therapy (Kim et al., 2022; Leenders et al., 2021), demonstrating the functional and clinical importance of tankyrase-mediated degradation of axin proteins.

Here, we used human cell lines to study a variant of phosphorylated axin (hereafter referred to as phospho-axin) that became apparent as distinct second band with reduced electrophoretic mobility on western blots. Axin was phosphorylated by casein kinase 1 alpha 1 (CSNK1A1, also known as CK1α) and dephosphorylated by the catalytic subunit alpha of protein phosphatase 1 (PPP1CA, hereafter referred to as PP1), and axin condensates favored phosphorylation by enriching CK1α over PP1. We then mapped the phosphorylation site to an N-terminal CK1 phosphorylation consensus motif overlapping with the tankyrase-binding site, which was not conserved in axin2. Importantly, axin phosphorylation sterically and/or electrostatically hindered tankyrase binding, counteracted tankyrase-mediated degradation and increased axin stability.

## RESULTS

### Phosphorylation of axin increases its stability

**Fig. 1.**
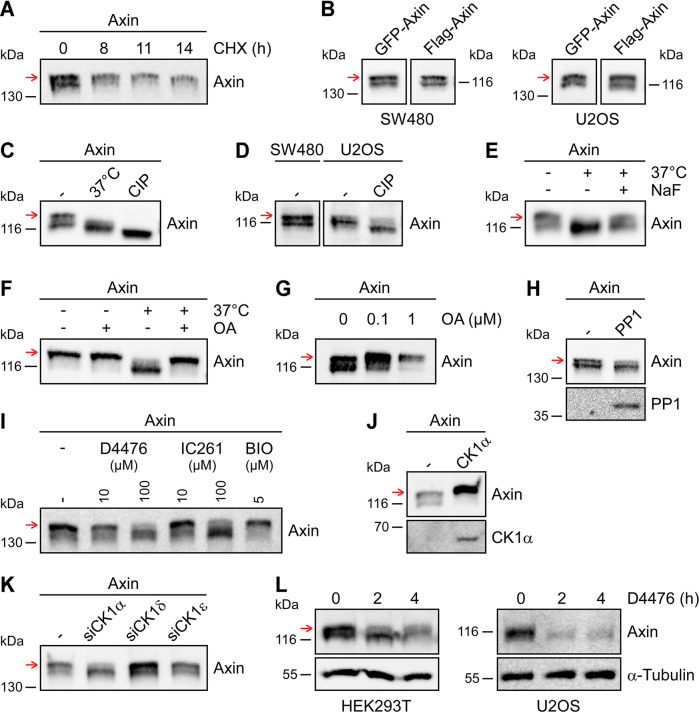
**Axin phosphorylation increases its stability.** (A-L) Representative western blots showing axin in hypotonic cell extracts. Red arrows indicate the phospho-axin variant. (A) HEK293T cells expressing GFP-tagged rat axin (GFP-axin) were left untreated (0) or were treated with 100 µM cycloheximide (CHX) for 8, 11 or 14 h. (B) GFP-axin or Flag-tagged rat axin (Flag-axin) expressed in SW480 and U2OS cells. (C) Flag-axin expressed in SW480 cells. Extracts were left untreated (–) or incubated at 37°C for 1 h without (37°C) or with calf intestinal phosphatase (CIP). (D) Endogenous human axin in SW480 (left) and U2OS cells (right). Extracts were untreated (–) or CIP-treated. (E) Flag-axin expressed in SW480 cells. Extracts were left untreated (–) or incubated at 37°C for 1 h without or with sodium fluoride (NaF, 10 mM). (F) Flag-axin expressed in SW480 cells. Extracts were supplemented with okadaic acid (OA, 1 µM) and/or incubated at 37°C, as indicated. (G) U2OS cells expressing Flag-axin were left untreated (0) or treated for 6 h with the indicated OA concentrations. (H) GFP-axin expressed alone (–) or together with Flag-tagged human PP1 (Flag-PP1) in U2OS cells. (I) U2OS cells expressing GFP-axin were left untreated (–) or treated for 6 h with CK1 inhibitors D4476 or IC261, or the GSK3 inhibitor BIO, as indicated. (J) Flag-axin expressed alone (–) or together with GFP-tagged human CK1α (GFP-CK1α) in U2OS cells. (K) GFP-axin transfected in U2OS cells either together with a control siRNA (–) or with siRNAs targeting human CK1α, CK1δ or CK1ε as indicated (siCK1α, siCK1δ, siCK1ε, respectively). (L) Endogenous axin in HEK293T and U2OS cells that were left untreated (0) or treated for 2 or 4 h with 100 µM D4476. α-Tubulin serves as loading control. All experiments were replicated at least three times.

**Fig. 2.**
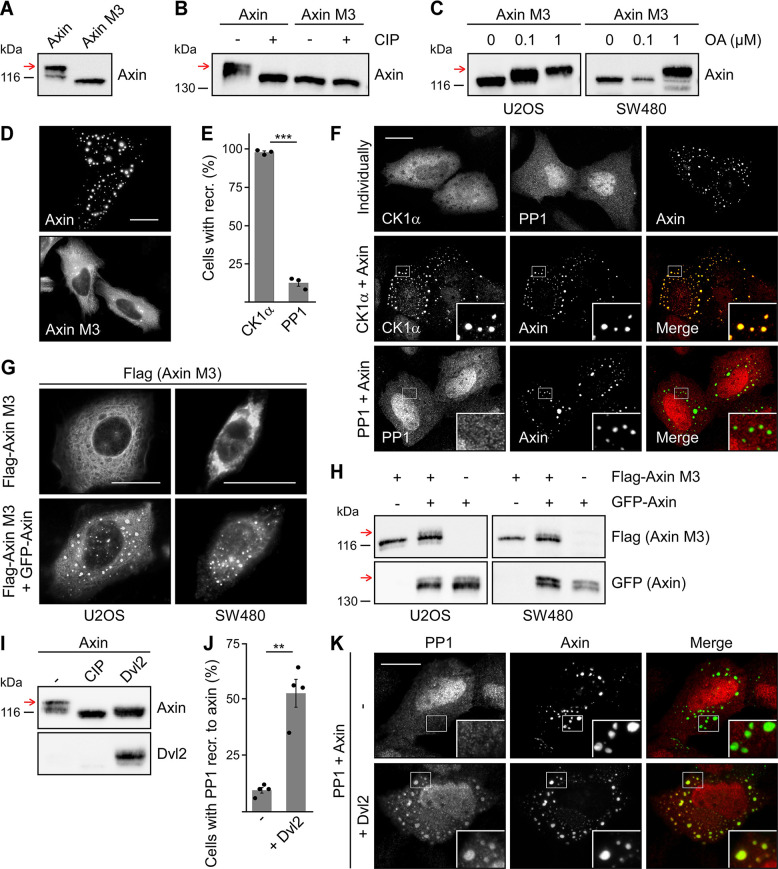
**Axin phosphorylation depends on condensates.** (A–C, H, I) Western blotting for axin and axin M3 in hypotonic cell extracts. Red arrows indicate the phospho-axin variant. (A) Flag-tagged rat axin (Flag-axin) and axin M3 (Flag-axin M3) expressed in SW480 cells. (B) GFP-tagged rat axin (GFP-axin) and axin M3 (GFP-axin M3) expressed in SW480 cells. Extracts were left untreated (–) or treated with calf intestinal phosphatase (CIP). (C) Flag-axin M3 expressed in U2OS and SW480 cells, which were left untreated (0) or treated for 6 h with okadaic acid (OA) at concentrations as indicated. (H) U2OS and SW480 cells expressing Flag-axin M3 and GFP-axin individually and in combination. (I) Extracts of U2OS cells expressing Flag-axin only that had been left untreated (–) or had been treated with calf intestinal phosphatase (CIP), and extracts of U2OS cells co-expressing Flag-axin together with CFP-tagged mouse Dvl2 (Dvl2). (D) Representative fluorescence images of U2OS cells expressing GFP-axin or GFP-axin M3. (E) Quantification of cells as described in F, showing recruitment (recr.) of CK1α or PP1 to the axin condensates as a percentage of 300 cells that co-express CK1α or PP1 (*n*=3). Results are the mean ±s.e.m.; ***P<0.001 (two-tailed, paired Student's *t*-test). (F) Representative immunofluorescence images of U2OS cells expressing GFP-axin, Flag-tagged human CK1α (Flag-CK1α) or Flag-tagged human PP1 (Flag-PP1) only (top), and of U2OS cells co-expressing GFP-axin with either Flag-CK1α (middle) or Flag-PP1 (bottom). Merged images are shown in color, with Flag immunofluorescence in red and GFP fluorescence in green. Insets (bottom right) are magnifications of the boxed areas. (G) Representative Flag immunofluorescence images of U2OS and SW480 cells expressing Flag-axin M3 alone (top) or together with GFP-axin (bottom). GFP fluorescence is not shown. (J) Quantification of cells as described in K, showing recruitment of PP1 to the axin condensates as a percentage of 400 cells that co-express PP1 in the absence (–) or presence (+) of Dvl2 (*n*=4). Results are the mean ±s.e.m.; **P<0.01 (two-tailed, paired Student's *t*-test). (K) Representative immunofluorescence images of U2OS cells that had been transfected with Flag-PP1 and GFP-axin (–) or with Flag-PP1, GFP-axin and HA-tagged mouse Dvl2 (+). Merged images are shown in color, with Flag immunofluorescence in red and GFP fluorescence in green. Insets (bottom right) are magnifications of the boxed areas. Scale bars: 20 µm (D, F, G, K). All experiments were replicated at least three times.

In cell culture assays with transiently expressed axin, we noticed a distinct double band for axin on western blots (Fig. 1A). The axin variant in the upper band exhibited a markedly increased band intensity compared to the variant in the lower band when translation was inhibited by cycloheximide (Fig. 1A). This observation suggested that the upper band variant is degraded more slowly than the lower band variant. The double band was consistently detectable in different human cell lines, including HEK293T, SW480 and U2OS (Fig. 1A,B); with different protein tags (Fig. 1B); and with axin proteins from different species, namely rat and human (Fig. 1A,D). Interestingly, the upper band was absent after incubation of cell extracts at 37°C, suggesting that the respective axin variant carried a post-translational modification that was unstable or reversible by endogenous enzymes (Fig. 1C). Similarly, treatment of cell extracts with calf intestinal phosphatase removed the upper axin band, providing first evidence that the investigated modification was a phosphorylation (Fig. 1C). Indeed, some types of phosphorylation markedly decrease the electrophoretic mobility of proteins, resulting in an increase in the molecular mass of the respective protein band on western blots by several kilodaltons, equalling to far more than the mass of the phosphate (Lee et al., 2019; Wegener and Jones, 1984). Interestingly, axin that had been treated with calf intestinal phosphatase migrated even faster than the lower band of the axin double band, indicating additional phosphorylation sites in axin (Fig. 1C), which is in line with previous studies (Jho et al., 1999; Luo et al., 2007; Yamamoto et al., 1999). As observed for transiently expressed rat axin (Fig. 2B,C), endogenous human axin also revealed a double band upon western blotting, with its upper band being lost upon treatment with calf intestinal phosphatase (Fig. 1D). Supplementing cell extracts with the non-specific Ser/Thr phosphatase inhibitor sodium fluoride (NaF) stabilized the upper axin band during incubation at 37°C, supporting the idea that it comprises a phospho-axin variant (Fig. 1E). The more-specific phosphatase inhibitor okadaic acid, which blocks protein phosphatase 2A (PP2A) and, at higher concentrations, PP1, was also able to protect axin from dephosphorylation in cell extracts (Fig. 1F). Western blotting of extracts obtained from living cells treated with okadaic acid showed a strongly increased upper axin band (Fig. 1G), suggesting that PP1 or PP2A dephosphorylated the respective phospho-axin variant in the absence of inhibitors. Consistently, co-expression of axin with PP1 strongly decreased the upper axin band identifying PP1 as a crucial phosphatase (Fig. 1H). To search for the opposing kinase, we inhibited the two predominant kinases of the Wnt pathway, i.e. CK1 and GSK3B. While inhibition of CK1 with D4476 or IC261 markedly decreased the upper axin band (Fig. 1I; Fig. S1A), inhibition of GSK3B with LiCl or BIO showed no significant effect (Fig. 1I; Fig. S1B). Consistently, co-expression of axin with CK1α or CK1ε (also known as CSNK1E) markedly increased the upper axin band (Fig. 1J; Fig. S1C). Knockdown experiments using small interfering RNAs (siRNAs) targeting different CK1 isoforms showed that knockdown of CK1α strongly decreased the upper axin band, whereas knockdown of CK1δ (also known as CSNK1D) or CK1ϵ did not, identifying CK1α as the crucial endogenous kinase (Fig. 1K; Fig. S1D). Together, our experiments suggest that phosphorylation of axin by CK1α generates a phospho-variant that is detected as distinct upper band by western blotting (red arrow), and that PP1 counteracts this phosphorylation. In line with a higher stability of this CK1α-phosphorylated axin variant in our cycloheximide experiments (Fig. 1A), CK1 inhibition by D4476 strongly decreased phosphorylation and, subsequently, expression of endogenous axin (Fig. 1L).

### Phosphorylation of axin depends on condensates

Interestingly, an axin DIX-domain mutant (hereafter referred to as axin M3) showed only a single band on western blots, its molecular mass being similar to that of calf intestinal phosphatase-treated, dephosphorylated wild-type (WT) axin, indicating that axin M3 was not phosphorylated (Fig. 2A,B). However, treatment of axin M3-expressing cells with okadaic acid resulted in a clear upshift of the axin M3 band (Fig. 2C), as observed similarly for WT axin (Fig. 1G). These findings suggested that axin M3 can be phosphorylated in principal but that dephosphorylation by PP1 prevailed in this mutant. The mutation in axin M3 inhibits head-to-tail polymerization of the axin DIX domain by inactivating the head interaction interface, thereby preventing the formation of axin condensates (Fig. 2D) (Fiedler et al., 2011). As biomolecular condensates are able to promote enzymatic reactions by enriching certain factors while excluding others (Banani et al., 2017), we hypothesized that axin condensates promote phosphorylation of axin and/or protect axin from dephosphorylation. Indeed, immunofluorescence-based experiments revealed that axin condensates recruited co-expressed CK1α almost completely, while co-expressed PP1 was almost absent from condensates (Fig. 2E,F), suggesting a phosphorylation-supportive environment inside condensates. Similarly to PP1, PP2A was not recruited into axin condensates (Fig. S2A,B). To test the hypothesis that axin condensates promote phosphorylation further, we investigated whether axin M3 is phosphorylated when recruited into condensates. For this, we co-expressed axin M3 together with WT axin. As anticipated, immunofluorescence analysis showed recruitment of axin M3 in WT axin condensates (Fig. 2G), most probably through the tail-interaction interface of the DIX domain, which is not affected by the M3 mutation (Fiedler et al., 2011). Importantly, a faint second band of axin M3 appeared above its regular band on western blots upon co-expression of WT axin, consistent with phosphorylation of axin M3 in WT axin condensates (Fig. 2H). We have previously shown that replacement of the regulator of G-protein signaling (RGS) domain in axin with that of axin2 results in a chimeric axin protein (cAxin^RGS^) with diffuse cellular distribution (Fig. S3A,B) because the axin2 RGS domain contains an aggregating protein sequence that prevents condensates (Bernkopf et al., 2019; Miete et al., 2022). It is noteworthy that cAxin^RGS^ showed no condensates and no phosphorylation (Fig. S3B,C, blue arrow). As observed for axin M3, okadaic acid treatment resulted in a distinct upshift of the cAxin^RGS^ western blot band, demonstrating that phosphorylation is principally possible (Fig. S3D, red arrow). Thus, two independent mutations that prevented the formation of condensates (axin M3 and cAxin^RGS^) also abolished phosphorylation, supporting a role of condensates in phosphorylation. Interestingly, the upper axin band also disappeared upon co-expression of the positive Wnt pathway regulator dishevelled 2 (DVL2, hereafter referred to as Dvl2), which is indicative of decreased phosphorylation (Fig. 2I). Like axin, Dvl2 contains a DIX domain, mediating the formation of condensates (Schwarz-Romond et al., 2007a, 2005). Axin and Dvl2 formed mixed condensates upon co-expression (Fig. S2C,E), as had been described previously (Schwarz-Romond et al., 2007b). Notably, incorporation of Dvl2 into axin condensates promoted recruitment of PP1 (Fig. 2J,K), potentially explaining how Dvl2 can induce dephosphorylation of axin within intact condensates. As PP1 was also enriched in Dvl2 condensates (Fig. S2D,F), Dvl2 might recruit PP1 to axin condensates. Taken together, the collective data suggest that axin condensates promote axin phosphorylation through enrichment of CK1α but exclusion of PP1.

### Axin is phosphorylated at an N-terminal CK1 motif

**Fig. 3.**
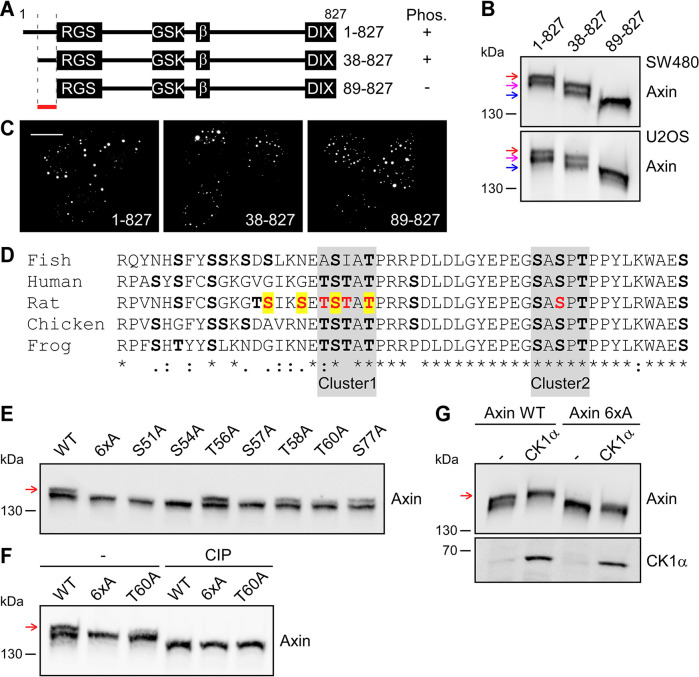
**Axin is phosphorylated at an N-terminal CK1 motif.** (A) Schematic of WT rat axin and two rat axin deletion constructs [GFP-axin 1-827 (1-827), GFP-axin 38-827 (38-827) and GFP-axin 89-827 (89-827), respectively], showing the aa regions that bind APC (RGS), GSK3B and β-catenin (GSK and β, respectively), or promote its polymerization (DIX). Presence (+) or absence (–) of phosphorylation (Phos.) according to the findings described in B is indicated. The phosphorylation site is located between aa residues 38 and 88 (red horizontal line). (B, E–G) Western blotting for axin and axin mutants in hypotonic cell extracts. Red arrows indicate the phospho-axin variant. (B) Indicated GFP-tagged axin constructs expressed in SW480 and U2OS cells. Magenta and blue arrow indicate the positions of the phospho-variant of axin 38-827 and the missing phospho-variant of axin 89-827, respectively. (E) GFP-tagged WT rat axin and the indicated GFP-tagged rat axin mutants expressed in U2OS cells. (F) GFP-tagged axin and indicated axin mutants expressed in SW480 cells. Extracts were untreated (–) or calf intestinal phosphatase-treated (CIP). (G) GFP-tagged axin and indicated axin mutants expressed in SW480 cells alone or together with GFP-tagged human CK1α (GFP-CK1α). (C) Fluorescence images of GFP-tagged full-length axin and deletion mutants axin 1-827, axin 38-827 and axin 89-827 in U2OS cells. Scale bar: 20 µm. (D) Clustal Omega alignment (according to Sievers et al., 2011) of axin sequences from zebrafish (*Danio rerio*, UniProt AC: P57094), human (*Homo sapiens*, UniProt AC: O15169), rat (*Rattus norvegicus*, UniProt AC: O70239), chicken (*Gallus gallus*, UniProt AC: O42400) and frog (*Xenopus laevis*, UniProt AC: Q9YGY0). Asterisks indicate identical aa residues. Dots and colons indicate weakly similar aa residue properties and strongly similar aa residue properties, respectively. Ser/Thr residues are shown in bold. Conserved Ser/Thr residues (i.e. cluster 1 and 2) are underlaid in grey. Ser/Thr residues analyzed through mutation are shown in red. Ser/Thr residues affecting the electrophoretic mobility are highlighted in yellow. All experiments were replicated at least three times.

To map the crucial site(s) within axin that are phosphorylated by CK1, we used axin mutants comprising N-terminal deletions, which did not compromise condensate formation through the C-terminal DIX domain (Fig. 3A). An axin protein lacking amino acid (aa) residues 1–37 (hereafter referred to as axin 38-827) showed a double band on western blots, which was highly reminiscent of the phosphorylation-dependent double band of WT axin (Fig. 3B, magenta arrows). In contrast, an axin protein lacking aa residues 1–88 (hereafter referred to as axin 89-827) showed only a single band, indicating that aa residues 38–88 are important for phosphorylation (Fig. 3B, blue arrows). Since axin 89-827 was still able to form condensates (Fig. 3C), aa residues 38–88 might contain the crucial phosphorylation site(s). CK1α is a serine/threonine (Ser/Thr) protein kinase. Alignment of axin protein sequences from different vertebrate species revealed two clusters of conserved Ser/Thr residues within aa residues 38–88 (Fig. 3D, grey boxes). Importantly, mutation of six Ser (S)/Thr (T) residues – i.e. S51, S54, T56, S57, T58 and T60 – next to and within cluster 1 (Fig. 3D, red) to alanine (yielding mutant axin 6xA) abolished the upper, phosphorylation-dependent axin band (Fig. 3E). Individual analysis of these residues revealed that S51, S54, S57 and T60 were crucial for the phosphorylation of axin, while T56 and T58 were dispensable (Fig. 3E). Noteworthy, the four crucial residues constitute a typical CK1 phosphorylation motif according to the consensus sequence S/TxxS/TxxS/TxxS/T (with x representing any aa) (Fig. 3D, yellow). A S77A mutation within cluster 2 (Fig. 3D, red) did not affect axin phosphorylation (Fig. 3E). Among the crucial residues, T60 was of special interest because phosphorylation of this residue is detected repeatedly in high-throughput analyses according to the PhosphoSitePlus database (Hornbeck et al., 2015). While axin 6xA and axin T60A showed single western blot bands – compared with the WT double band, all three proteins showed single bands at the same height after phosphatase treatment, suggesting that the initial difference was, indeed, due to phosphorylation (Fig. 3F). Moreover, co-expression of CK1α did not – 6xA in contrast to WT axin – result in an upshift of axin (Fig. 3G). Thus, the upper axin band in western blots strictly depended on Ser/Thr residues within a CK1 phosphorylation consensus motif, strongly indicating that this axin variant is, indeed, phosphorylated by CK1α at these residues.

### N-terminal phosphorylation by CK1α is not conserved in axin2

The alignment of mammalian axin and axin2 protein sequences revealed that none of the four identified crucial Ser/Thr residues is evolutionary conserved in axin2 (Fig. S4A). Consistently, axin2 exhibited only a single band on western blots, which migrated at the same height as the band of its M3 mutant and which was not affected by phosphatase treatment, showing no indication of phosphorylation (Fig. S4B,C, blue arrow). Axin2 does not form condensates (Behrens et al., 1998; Bernkopf et al., 2019), which, potentially, explains the absence of phosphorylation (Fig. S4D). However, in contrast to axin M3 and cAxin^RGS^ (Fig. 2C; Fig. S3D), treatment with okadaic acid did not result in a second axin2 band (Fig. S4E), suggesting that phosphorylation of axin2 was impossible. Consistently, replacing axin aa residues 1–75, which contain the CK1 phosphorylation sites, with the respective axin2 sequence (cAxin^1-75^) completely abolished phosphorylation (Fig. S4F,G, blue arrow), although condensate formation was still intact (Fig. S4D). Together, our data strongly suggest that the discovered phosphorylation by CK1α at an N-terminal consensus motif is isoform-specific for axin and functionally not conserved in axin2.

### Phosphorylation at Thr60 counteracts tankyrase-mediated degradation of axin

**Fig. 4.**
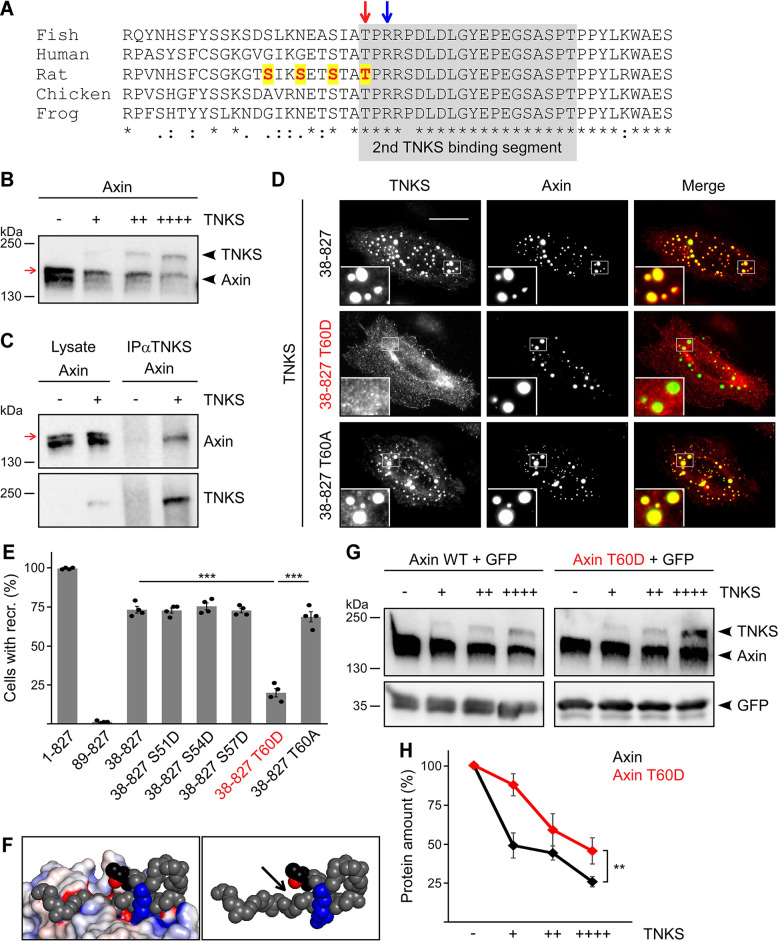
**Axin phosphorylation counteracts tankyrase-mediated degradation.** (A) Shown is the same Clustal Omega alignment of axin sequences as in Fig. 3D, but with the second tankyrase (TNKS)-binding segment underlaid in grey (Morrone et al., 2012). The red arrow indicates Thr (T) 60, phosphorylation of which impairs tankyrase binding (see below). The blue arrow indicates Arg (R) 62, a crucial residue that mediates tankyrase binding (Morrone et al., 2012). Asterisks indicate identical aa residues. Dots and colons indicate weakly similar aa residue properties and strongly similar aa residue properties, respectively. Ser/Thr residues analyzed through mutation are shown in red. Ser/Thr residues affecting the electrophoretic mobility are highlighted in yellow. (B) Western blotting of transiently expressed GFP-tagged rat axin (GFP-axin) and GFP-tagged human tankyrase (GFP-tankyrase) in extracts of HEK293T cells. Red arrow indicates the phospho-axin variant. –, 0 ng; +, 62.5 ng; ++, 125 ng; ++++, 250 ng per 2×10^5^ cells. (C) Western blotting for axin and tankyrase in lysates of U2OS cells (lysate) expressing GFP-axin alone (−) or together with Flag-tagged human tankyrase (Flag-tankyrase, +), and after immunoprecipitation from these lysates using an anti-Flag antibody (IPαTNKS). Red arrow indicates the phospho-axin variant. (D) Representative immunofluorescence images of U2OS cells expressing Flag-tankyrase together with one of the GFP-axin mutants as indicated on the left. Merged images are shown in color, with Flag immunofluorescence in red and GFP fluorescence in green. Insets (bottom left) are magnifications of the boxed areas. Scale bar: 20 µm. (E) Quantification of cells co-expressing Flag-tankyrase together with either GFP-axin (1-827) or one of the GFP-tagged axin mutants as indicated (see Fig. S5A for respective representative immunofluorescence images), showing recruitment (recr.) of tankyrase to the axin condensates as a percentage of 400 cells (*n*=4). Results are the mean±s.e.m.; ***P<0.001 (two-tailed, paired Student's *t*-test). Condensates of GFP-axin 38-827 T60D (shown in red) exhibited significant defects in tankyrase recruitment (see also panel D, middle row). (F) Structure of axin–tankyrase interaction, showing the second tankyrase-binding segment as described by Morrone et al., (2012). The axin main chain (grey), Thr60 (black; main and side chain), its phosphorylatable oxygen atom (red) and Arg62 (blue; main and side chain) are depicted as spheres. The electrostatic potential surface of tankyrase is shown on the left and hidden on the right. (G) Western blotting for GFP in extracts of HEK293T cells transiently co-expressing GFP and GFP-tankyrase together with either GFP-axin WT or GFP-axin T60D, as indicated. –, 0 ng; +, 62.5 ng; ++, 125 ng; ++++, 250 ng per 2×10^5^ cells. GFP serves as expression and loading control. (H) Quantification of the amount of WT axin (black) and axin T60D (red) in percent. Axin amounts were determined by 2D densitometry and normalized to GFP of three independent experiments as described for panel G (*n*=3). Results are the mean±s.e.m.; **P<0.01 (two-tailed, paired Student's *t*-test). Axin T60D was significantly more stable than WT axin in the presence of tankyrase. All experiments were replicated at least three times.

**Fig. 5.**
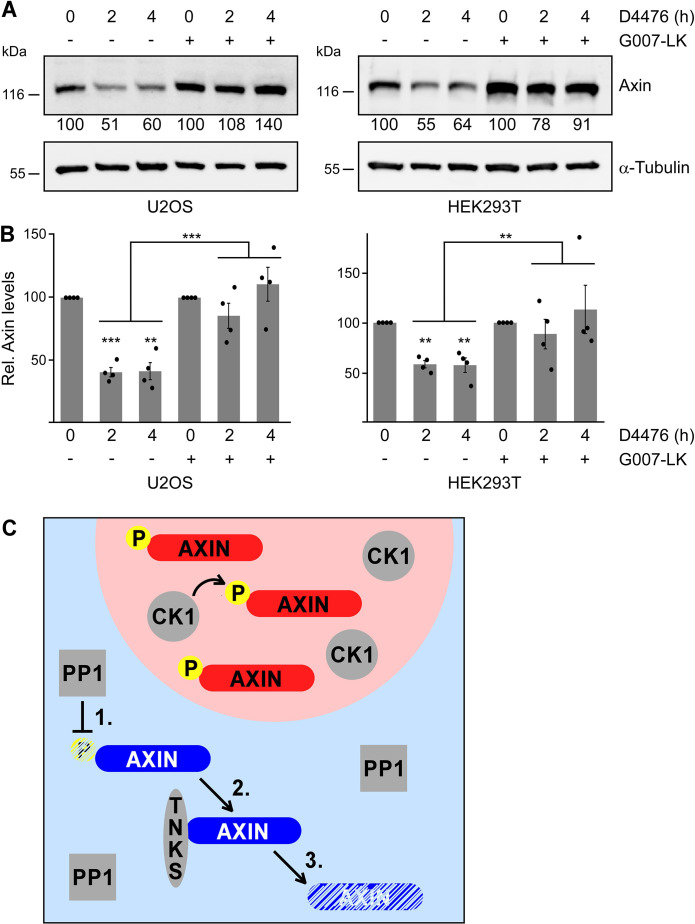
**Axin phosphorylation in condensates counteracts tankyrase-mediated degradation.** (A) Western blotting for endogenous axin in lysates of U2OS (left) and HEK293T cells (right). Cells had been pre-treated overnight with the tankyrase inhibitor G007-LK (500 nM) (+) or not (−), and were then treated with 100 µM CK1 inhibitor D4476 for 2 h or 4 h, or left untreated (0). α-Tubulin serves as loading control. Numbers below the blots show 2D densitometry quantification of axin bands normalized to α-tubulin. To compare the effect of D4476 in cells pre-treated with G007-LK or not, the initial amount of axin in untreated cells (0 h D4476) was set to 100% for either pre-treatment, i.e. the amount of axin in D4476-treated cells is presented relative to the respective initial amount. (B) Quantification of the experiments shown in A (*n*=4). Results are the mean±s.e.m.; **P<0.01, ***P<0.001 (two-tailed, paired Student's *t*-test). (C) Schematic depicting how phosphorylation of axin prevents its degradation through tankyrase. Within condensates (light red background): CK1α (CK1) phosphorylates axin at its N-terminal consensus motif, thereby sterically hindering tankyrase binding and increasing axin stability. Outside condensates (light blue background): PP1 dephosphorylates axin (1), thereby allowing tankyrase (TNKS) binding (2) and promoting tankyrase-mediated degradation of axin (3).

Since tankyrase promotes axin degradation (Huang et al., 2009; Morrone et al., 2012) and binds axin in close proximity to the identified CK1 phosphorylation cluster (Fig. 4A), phosphorylation at these residues might protect axin from tankyrase-mediated degradation. To test this hypothesis, we performed axin degradation assays. Co-expression of axin with tankyrase induced degradation of axin in a tankyrase dose-dependent manner (Fig. 4B). Importantly, the phospho-axin variant (upper band) was markedly more stable compared to the unphosphorylated axin variant (lower band) in the presence of tankyrase (Fig. 4B), similarly as seen after treatment with cycloheximide (Fig. 1A). Consistently, co-immunoprecipitation experiments suggested that tankyrase preferentially interacts with unphosphorylated axin (Fig. 4C, lower band). Interaction of axin with tankyrase could also be studied by using immunofluorescence assays, as axin recruited tankyrase in condensates (Fig. S5A). Axin contains two tankyrase-binding segments in the N-terminus between aa residues 18–30 and 60–79 (Morrone et al., 2012). Deletion of both tankyrase-binding segments in axin (axin 89-827) completely abolished recruitment of tankyrase into condensates, demonstrating the specificity of the assay (Fig. 4E; Fig. S5A). Interestingly, the second tankyrase-binding segment was sufficient to mediate axin–tankyrase interaction in this assay because axin 38-827, which contains the second tankyrase-binding segment only, still recruited tankyrase (Fig. 4A,D,E). To investigate whether phosphorylation of the CK1 sites impairs axin–tankyrase interaction, phospho-mimicking mutants were generated by replacing the identified Ser/Thr residues with aspartate (Asp/D) to permanently mimic the negative charge of the phosphate (S51D, S54D, S57D and T60D). Axin 38-827 S51D, axin 38-827 S54D and axin 38-827 S57D showed no decrease in tankyrase recruitment compared to axin 38-827, suggesting that phosphorylation of S51, S54 or S57 does not impair axin–tankyrase binding (Fig. 4E; Fig. S5A). In contrast, axin 38-827 T60D did show significant defects in tankyrase recruitment (Fig. 4D,E). For this, the negative charge and/or the size of the Asp appeared to be crucial because axin 38-827 T60A recruited tankyrase as efficiently as axin 38-827, strongly suggesting that phosphorylation of T60 attenuates tankyrase binding (Fig. 4D,E). Consistently, co-expression of CK1 almost completely abolished recruitment of tankyrase to axin 38-827 (Fig. S5B,C). According to the crystal structure of axin–tankyrase binding (Morrone et al., 2012), the second tankyrase-binding segment of axin forms a loop that is essential to optimally align the crucial arginine residue at position 62 (Arg62) for tankyrase binding (Fig. 4A,F). A negatively charged, bulky phosphate group at Thr60 might sterically interfere with this loop formation and/or prevent correct alignment of the close-by crucial Arg62 by electrostatic attraction (Fig. 4A,F). Next, we studied the impact of T60 phosphorylation on tankyrase-mediated degradation in the context of full-length proteins through phospho-mimicking. Importantly, upon tankyrase co-expression, axin T60D showed significantly increased stability compared to WT axin (Fig. 4G,H), indicating that phosphorylation of T60 can counteract degradation of axin through tankyrase. In line with this, tankyrase inhibition rescued the decrease of endogenous axin levels upon CK1 inhibition (Fig. 5A,B). Based on the data presented here, we propose that phosphorylation of T60 by CK1α attenuates axin–tankyrase interaction, thereby counteracting tankyrase-mediated degradation of axin (Fig. 5C).

## DISCUSSION

In this study, we discovered that phosphorylation of axin by CK1α counteracts tankyrase-mediated axin degradation (Fig. 5C). Consistently, CK1-phosphorylated axin exhibited increased stability when translation was inhibited by cycloheximide (Fig. 1A) and when tankyrase was co-expressed in degradation assays (Fig. 4B). Moreover, CK1 inhibition markedly decreased the expression levels of endogenous axin (Fig. 1L) and inhibition of tankyrase rescued this decrease (Fig. 5A,B), which is in line with tankyrase-mediated degradation of dephosphorylated axin. The crucial phosphorylation site could be mapped to Thr60. Importantly, a phosphorylation-mimicking mutation of Thr60Asp (T60D) impaired axin–tankyrase interaction (Fig. 4D,E) and increased axin stability in degradation assays when tankyrase was co-expressed (Fig. 4G,H). Thr60 is located within the second tankyrase-binding segment of axin (Fig. 4A). Structural analysis indicated that a phosphate group on Thr60 electrostatically and/or sterically hinders the folding of axin that is required for tankyrase binding (Fig. 4F), suggesting a molecular mechanism of how phosphorylation counteracts degradation. Thr60 is part of a CK1 phosphorylation consensus motif (SxxT), which is evolutionary conserved in vertebrates from mammals to amphibians, fish and birds (Fig. 3D). Phosphorylation of axin Thr60 has been detected in 16 high-throughput analyses, rendering it one of the top five phosphorylated residues in axin according to the PhosphoSitePlus database (Hornbeck et al., 2015), although the function of this phosphorylation has, until now, remained unknown. The high detection frequency suggests that phosphorylation of Thr60 occurs rather frequently and, most probably, to protect a certain pool of axin from tankyrase-mediated degradation. Based on the collective data, we propose that phosphorylation of axin by CK1 contributes to inhibition of the Wnt/β-catenin signaling pathway by stabilizing the scaffold protein that mediates phosphorylation and subsequent degradation of β-catenin.

Our data strongly suggest that phosphorylation of axin crucially depends on the formation of axin condensates because axin M3 and cAxin^RGS^ – axin mutants that cannot form condensates due to mutations that are position- and concept-wise unrelated – also lack phosphorylation (Fig. 2A,B,D; Fig. S3B,C), pointing to a causative link between condensates and phosphorylation. Moreover, following the partial rescue of condensate formation upon recruitment of axin M3 into condensates of WT axin, phosphorylation was partially rescued as well (Fig. 2G,H). Biomolecular condensates can promote distinct cellular reactions by enriching crucial reaction factors and/or excluding antagonizing factors (Banani et al., 2017). In case of axin phosphorylation, we observed that axin condensates strongly recruited CK1α, whereas the antagonizing phosphatase PP1 was almost absent (Fig. 2E,F). Thus, axin condensates are most likely to promote phosphorylation by enriching the required kinase over the antagonizing phosphatase. Loss of condensate formation would increase dephosphorylation, as axin becomes more accessible for PP1. In line with this hypothesis, inhibition of the phosphatase was able to rescue phosphorylation of the mutants axin M3 and cAxin^RGS^, which cannot form condensates (Fig. 2C; Fig. S3D). Taken together, axin condensates seem to promote phosphorylation and, thus, stabilization of axin, whereas axin outside condensates is dephosphorylated by PP1 and degraded through tankyrase (Fig. 5C). This mechanism might actively promote the formation of phase-separated axin condensates in an anticipating feedforward manner, as it stabilizes and destabilizes axin in the phases of high and low protein concentration, respectively. Stabilization of phase-separating biomolecules inside condensates and their destabilization outside would offer an elegant feedforward mechanism to support phase separation in general, and might have implications for the self-organization of biomolecular condensates beyond axin.

Interestingly, the positive Wnt pathway regulator Dvl2 abolished axin phosphorylation (Fig. 2I) without dissolving axin condensates (Fig. S2C,E). Similar to axin, Dvl2 contains a DIX domain allowing homopolymerization and heteropolymerization with axin (Fiedler et al., 2011; Schwarz-Romond et al., 2007a,b). Importantly, incorporation of Dvl2 in axin condensates promoted recruitment of PP1 (Fig. 2J,K), potentially explaining how Dvl2 abolished phosphorylation of axin within intact condensates. PP1 also colocalized with homotypic Dvl2 condensates (Fig. S2D,F), indicating that Dvl2 may recruit PP1 to axin by interacting with both proteins. Alternatively or in addition, changes in the properties of axin condensates upon incorporation of Dvl2, such as increased dynamics (Schwarz-Romond et al., 2007b), may alter the selectivity of the condensates and allow recruitment of PP1. According to our proposed mechanism, dephosphorylation of axin by Dvl2 would destabilize axin, thereby contributing to activation of the Wnt pathway.

The presented insights of how condensates regulate axin phosphorylation might have broader implications for Wnt signaling, since the key function of axin condensates is to promote phosphorylation of β-catenin. In addition to providing a high avidity for components of the β-catenin destruction complex (Fiedler et al., 2011), axin condensates might promote phosphorylation of β-catenin by enriching kinases over antagonizing phosphatases, as seen for axin phosphorylation (Fig. 2E,F). Consistent with this idea, APC, which is known to induce formation of axin condensates (Kunttas-Tatli et al., 2014; Mendoza-Topaz et al., 2011), has been described to protect β-catenin from dephosphorylation (Su et al., 2008). It is noteworthy that a mechanism similar to that suggested for dephosphorylation of axin might contribute to dephosphorylation and subsequent stabilization of β-catenin by Dvl2, as axin-bound β-catenin might also become more accessible for phosphatases in heterotypic axin–Dvl2 condensates.

N-terminal phosphorylation by CK1 is not conserved for the closely related axin homolog axin2 (Fig. S4). This is interesting because tankyrase can degrade both axin homologs (Huang et al., 2009). The identified phosphorylation will, thus, allow to uncouple tankyrase-mediated degradation of axin and axin2 by exclusively protecting axin from degradation. This might be beneficial to, for example, reset axin2-mediated feedback inhibition of the Wnt pathway without impairing axin-mediated basal inhibition.

Small-molecule inhibitors of tankyrase have been investigated for targeted cancer therapy, as they potently inhibit the pathological Wnt signaling activity in colorectal cancer cells by stabilizing axin and axin2 (Huang et al., 2009). Although clinical trials on first-class tankyrase inhibitors have been limited by increased toxicity (Kim et al., 2022), new tankyrase inhibitors comprising increased affinity and selectivity have been developed to overcome these limitations (Kim et al., 2022; Leenders et al., 2021). As the mechanism described in our study regulates tankyrase-mediated degradation of axin, it might become clinically relevant at some point.

## MATERIALS AND METHODS

### Cell culture, transfections and chemicals

Cells of the human cell lines HEK293T, SW480 and U2OS were cultivated in Dulbecco's modified Eagle Medium supplemented with fetal bovine serum and antibiotics at 37°C in a humidified 10% CO_2_ atmosphere, according to ATCC recommendations. The cell lines had originally been obtained from ATCC and tested negative for mycoplasma contamination. Plasmid transfections were performed using polyethylenimine (U2OS and HEK293T cells) and Lipofectamine 2000 (SW480, Invitrogen, Thermo Fisher Scientific, Waltham, MA) and transfection of small interfering RNAs (siRNAs) was performed with Oligofectamine (Thermo Fisher Scientific). The CK1 inhibitors D4476 (ab120220) and IC261 (ab145189) were obtained from Abcam (Cambridge, UK) the GSK3 inhibitor BIO (B1686), the PP1 and PP2A inhibitor okadaic acid (O7885), the tankyrase inhibitor G007-LK (5.04907) and the protein synthesis inhibitor cycloheximide (239765) from Sigma-Aldrich (Merck, St. Louis, MO). For treatment of cells, the inhibitors were added to the cell culture medium. Inhibitor concentrations and treatment times are stated in the figure legends.

### Knockdown experiments

Cells were seeded and transfected the following day with a combination of plasmid DNA and siRNA, as indicated in the relevant figure legends, using Lipofectamine 2000 according to the instructions of the manufacturer (Invitrogen, Thermo Fisher Scientific, Waltham, MA). siRNA sequences are provided in the ‘Antibodies and siRNAs’ section below. The cells were lysed 48 h after transfection and subjected to western blotting (see below).

### Western blotting

Cells were seeded, transfected the following day, when required, and lysed at day three in hypotonic lysis buffer (20 mM Tris-HCl pH 7.5, 1 mM EDTA, Roche protease inhibitor cocktail). Treatments of cells or cell extracts are stated in the figures and figure legends. For calf intestinal phosphatase (CIP) treatment, cell extracts were treated at 37°C for 1 h with 0.5 U/µl CIP (New England Biolabs, Ipswich, MA). Extracts referred to as ‘untreated' in the figure legends were kept at 4°C on ice, as routinely done during cell lysis. For immunoprecipitation (Fig. 4C), Flag-TNKS and associated proteins were precipitated with α Flag antibodies on G/A beads (Santa Cruz Biotechnology, Dallas, TX), washed on beads and eluted by denaturation. Samples were denatured, and proteins separated by size using polyacrylamide gel electrophoresis. For separation of the upper and lower axin band 8% polyacrylamide gels were used. After protein transfer onto a nitrocellulose membrane (VWR International, Radnor, PA), the blots were probed with appropriate primary and horseradish peroxidase-conjugated secondary antibodies (see below). Protein bands were detected by using light emission upon horseradish peroxidase-catalyzed oxidation of luminol in a LAS-3000 with Image Reader software (FUJIFILM, Minato, Tokyo, Japan), and band intensities were quantified with AIDA 2D densitometry, when required. Uncropped blot images are provided for blot transparency (Fig. S6).

### Immunofluorescence

Cells were seeded, transfected the following day and stained on day three. For this, cells were fixed in ice-cold methanol (Figs 2D,G, 3C, 4D; Figs S2E, S3B, S4D, S5A,B) or 3% paraformaldehyde (Fig. 2F,K; Fig. S2B,F), permeabilized with 0.5% Triton X-100, blocked with cell culture medium, and incubated with appropriate primary and fluorochrome-conjugated secondary antibodies (see below). Analysis and image acquisition was performed at an Axioplan II microscope system (Carl Zeiss, Oberkochen, Germany) using a Plan-NEOFLUAR 100×/1.30 NA oil objective and a SPOT RT Monochrome camera (Diagnostic Instruments, MI).

### Molecular biology

Axin, axin2 or dishevelled constructs, conjugated to green fluorescent protein (GFP), cyan fluorescent protein (CFP) or the Flag-tag, were: GFP-axin (1-827; rat, as for all following axin mutants), GFP-axin M3, Flag-axin, Flag-axin M3, GFP-axin 89-827, GFP-cAxin^RGS^ (GFP-AC69-196), GFP-cAxin^DIX^ (GFP-AC700-840), GFP-cAxin^1-75^ (GFP-AC1-68), GFP-axin2 (GFP-Conductin, mouse), GFP-axin2 M3 (GFP-Conductin M3, mouse), CFP-Dvl2 (mouse) and HA-Dvl2 (mouse), as previously described (Bernkopf et al., 2019, 2015, 2018; Rauschenberger et al., 2017). GFP-axin 38-827, GFP-CK1α (human), Flag-CK1α (human), CK1ε, Flag-PP1 (human), GFP-TNKS (human tankyrase 1), Flag-TNKS (human tankyrase 1), Flag-PP2A-C (human), Flag-PP2A-Aα (human) and Flag-PP2A-Aβ (human) were cloned using standard molecular biology methods. For Flag-PP2A-C, Flag-PP2A-Aα and Flag-PP2A-Aβ, the Addgene plasmids #10689, #10884 and #15248 were used as templates (Chen et al., 2005, 2004; Sablina et al., 2007). GFP-axin 6xA, GFP-axin S51A, GFP-axin S54A, GFP-axin T56A, GFP-axin S57A, GFP-axin T58A, GFP-axin T60A, GFP-axin T60D, GFP-axin S77A, GFP-axin 38-827 S51D, GFP-axin 38-827 S54D, GFP-axin 38-827 S57D, GFP-axin 38-827 T60D, GFP-axin 38-827 T60A were generated using site directed mutagenesis. All newly generated expression vectors were verified by sequencing. Plasmids can be obtained from the corresponding author.

### Antibodies and siRNAs

Primary antibodies were rabbit anti-axin1 [western blotting (WB): 1:1000; cat. no.: 2087S, Cell Signaling Technology]; rabbit anti-phosphorylated-β-catenin (WB: 1:1000; cat. no.: 2009S, Cell Signaling Technology), mouse anti-GFP (WB: 1:1000; cat. no.: 11814460001, Merck), rat anti-HA High Affinity [immunofluorescence (IF): 1:800; cat. no.: 11867423001, Merck], rat anti-α-tubulin (WB: 1:1000; cat. no.: MCA77G, Bio-Rad), rabbit anti-Flag (IF: 1:400, WB: 1:1000; cat. no.: F7425, Sigma-Aldrich/Merck).

Antibodies against endogenous proteins (anti-axin1 and anti-phosphorylated β-catenin) were validated at our laboratory by knockdown experiments. Antibodies against tags (anti-GFP, anti-HA, anti-Flag) were validated by the absence of signal without the transient expression of tagged proteins.

Secondary antibodies were goat anti-rabbit-Cy3 (1:300), goat anti-rat-Cy2 (1:200) goat anti-mouse/rabbit/rat-HRP (between 1:1000 and 1:2000), all obtained from Jackson ImmunoResearch.

siRNAs targeting human CK1α (CSNK1A1; 5′-GCGAUGUACUUAAACUAUU-3′), CK1δ (CSNK1D; 5′-CGACCUCACAGGCCGACAA-3′) and CK1ε (CSNK1E; 5′-CCUCCGAAUUCUCAACAUA-3′) have been described previously (Cheong et al., 2015).

### Quantitative RT-PCR

Cells were lysed 48 h after siRNA transfection, total RNA was extracted using the RNeasy Mini Kit following the manufacturer's instructions (QIAGEN, Venlo, Netherlands) and transcribed into cDNA (AffinityScript QPCR cDNA Synthesis Kit, Agilent Technologies, Santa Clara, CA). The relative abundance of CK1 transcripts, i.e. of *CSNK1A1* (CK1α), *CSNK1D* (CK1δ), *CSNK1E* (CK1ε) and *GAPDH* (housekeeping gene) was determined by SYBR Green-based qPCR (CFX96 Real-Time System, Bio-Rad, Hercules, CA) with gene-specific primer pairs in technical triplicates (*CSNK1A1*: 5′-ATGGGTATTGGGCGTCACTG-3′, 5′-CCTGAGAAAGATGGGTCCTGAG-3′; *CSNK1D*: 5′-CCGCCATGGAGCTGAGAG-3′, 5′-ATGGCAACCTCTTCTCCTGC-3′; *CSNK1E*: 5′-GAGCTACGTGTGGGGAACAA-3′, 5′-TTGATGGCGACTTCCTCACC-3′; *GAPDH*: 5′-GTCAAGGCTGAGAACGGGAAGC-3′, 5′-GGACTCCACGACGTACTCAGCG-3′). The expression of the CK1 isoforms is presented relative to GAPDH.

### Sequence alignment

For the alignment of protein sequences, the Clustal Omega multiple sequence alignment program was used (Sievers et al., 2011). The UniProt accession numbers of all protein sequences are provided in the respective figure legend, allowing unambiguous identification.

### Structure analysis

The crystal structure of the axin–tankyrase complex (Morrone et al., 2012) was analyzed and visualized using PyMOL (version 1.8. Schrödinger, LLC). The electrostatic potential surface of tankyrase was determined using the Adaptive Poisson-Boltzmann Solver (Jurrus et al., 2018).

### Statistics

To probe the datasets for statistical significance two-tailed Student's *t*-tests were performed in a paired fashion owing to the experimental setup. Based on the nature of the assays and graphical assessment, we assumed normal distribution of the data, which was not formally tested owing to the small sample sizes. All *n* values are explicitly stated in the figure legends, and statistical significance is indicated by asterisks in the figures (*P<0.05, **P<0.01, ***P<0.001), when required. Sample sizes between three and four were chosen depending on the differences in exploratory experiments and the variability of the assay. The sample sizes are within the range found in published literature with similar methodologies, and no data of decent experimental quality was excluded. Samples, i.e. seeded cells, were allocated randomly into experimental groups. The investigator was blinded when quantifying phenotypes at the microscope (Figs 2E,J and 4E; Fig. S5C).

## Supplementary Material

10.1242/joces.261214_sup1 Supplementary information
