# Supplementary material for: Phosphorylation of axin within biomolecular condensates counteracts its tankyrase-mediated degradation
Source: J Cell Sci. 2023 Oct 27;136(20):jcs261214. doi: 10.1242/jcs.261214 (PMC10652037; doi:10.1242/jcs.261214)
Supplement: Supplementary information [file joces-136-261214-s1.pdf]

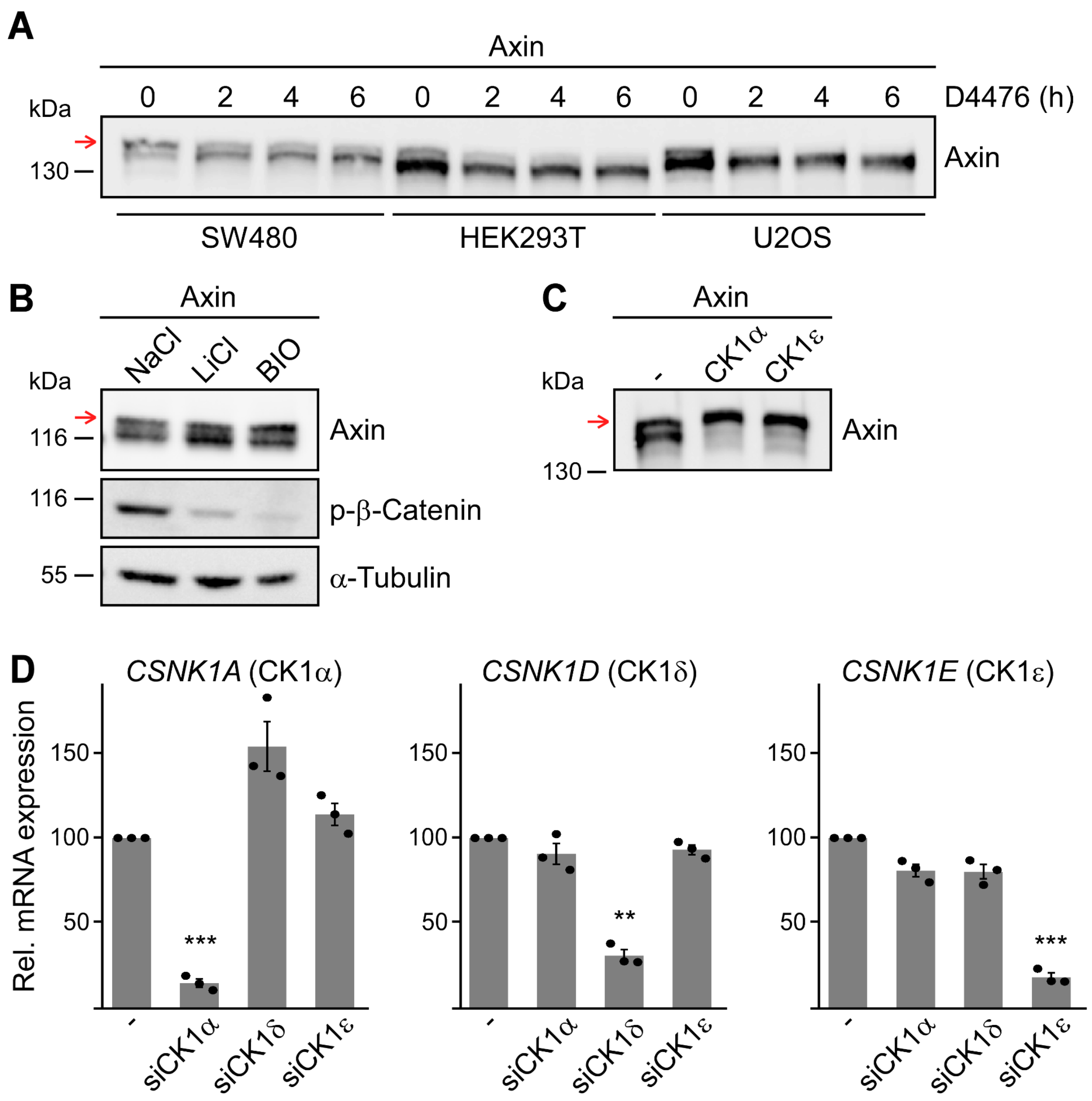

Fig. S1. Inhibition of CK1 and not of GSK3 reduced phosphorylation. A-C Western blotting for axin in hypotonic cell extracts. Red arrow indicates phospho-axin. **A** SW480, HEK293T and U2OS cells expressing GFP-axin were treated with 100 µM D4476 (CK1 inhibition) for 0, 2, 4 and 6 hours. **B** SW480 cells expressing Flag-axin were treated with 30 mM sodium chloride (control), 30 mM lithium chloride (GSK3 inhibition) or 5 µM BIO (GSK3 inhibition) for 6 hours. The marked decrease of GSK3-phosphorylated β-catenin (p-β-catenin) demonstrates efficient GSK3 inhibition. α-Tubulin serves as loading control. **C** GFP-axin expressed in U2OS cells either alone (-), or together with Flag-CK1α or with untagged CK1ε. **D** Relative mRNA expression of *CSNK1A* (encoding for CK1α), *CSNK1D* (encoding for CK1δ) and *CSNK1E* (encoding for CK1ε) normalized to the expression of the house keeping gene *GAPDH* in U2OS cells, which were transfected with a control siRNA (-) or with siRNAs targeting CK1α, δ or ε. Three independent experiments were performed in parallel with the experiments in Fig. 1K to demonstrate specificity and efficacy of the siRNA-mediated knockdown (n=3). Results are mean +- s.e.m., ** p<0.01, *** p<0.001. All experiments were replicated at least three times.

Journal of Cell Science • Supplementary information

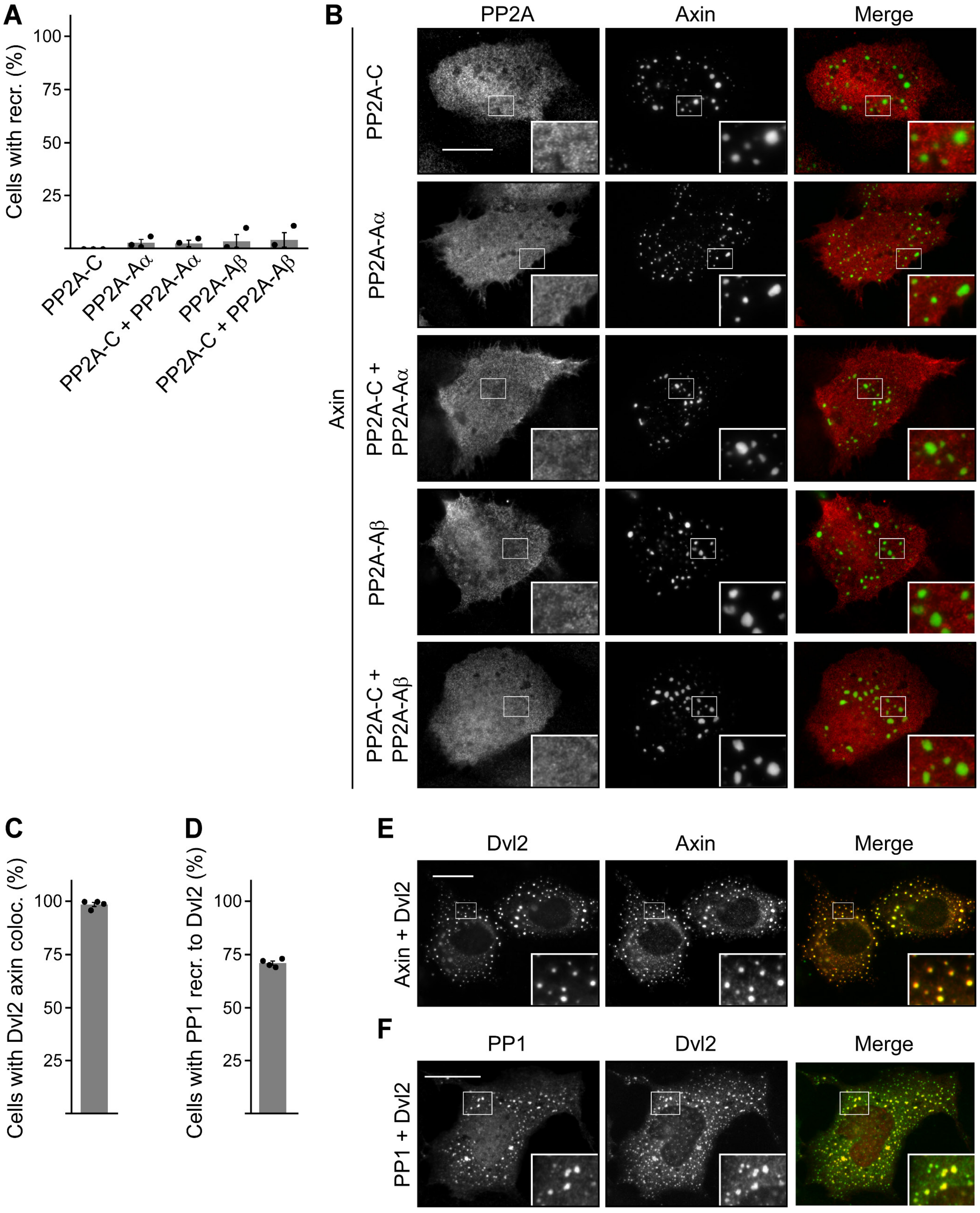

Journal of Cell Science • Supplementary information

Fig. S2. Analysis of the recruitment of phosphatases (PP2A and PP1) into axin or Dvl2 condensates. A Percentage of cells with recruitment of PP2A subunits in axin condensates out of 300 cells exhibiting axin condensates and co-expression of the PP2A subunits either alone or in combinations from three independent experiments, as described in B (n=3). Results are mean +- s.e.m. **B** Immunofluorescence staining of Flag (red) and GFP fluorescence (green) in U2OS cells expressing Flag-PP2A-C (catalytic subunit), Flag-PP2A-Aα (regulatory subunit Aα) and Flag-PP2A-Aβ (regulatory subunit Aβ) either alone or in combinations together with GFP-axin, as indicated on the left. **C** Percentage of cells with co-localization of Dvl2 and axin out of 400 cells exhibiting axin condensates and co-expression of Dvl2 from four independent experiments, as described in E (n=4). **D** Percentage of cells with recruitment of PP1 in Dvl2 condensates out of 400 cells exhibiting Dvl2 condensates and co-expression of PP1 from four independent experiments, as described in F (n=4). Results are mean +- s.e.m. (C,D). **E** CFP-Dvl2 fluorescence (green) and immunofluorescence staining of Flag-axin (red) in U2OS cells co-expressing both proteins. **F** Immunofluorescence staining of Flag-PP1 (red) and HA-Dvl2 (green) in U2OS cells co-expressing both proteins. Insets are magnified at the lower right; scale bars: 20 µm (B,E,F).

Journal of Cell Science • Supplementary information

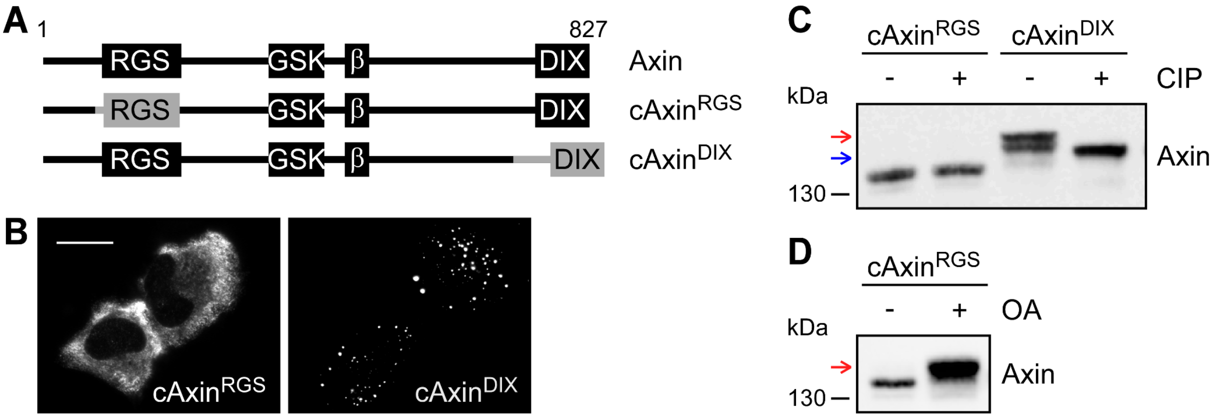

Fig. S3. An axin mutant lacking condensate formation lacked phosphorylation. A To scale schemes of axin and chimeric axin/axin2 proteins. Parts of axin and axin2 are shown in black and grey, respectively. **B** Fluorescence of indicated GFP-tagged proteins in U2OS cells. Scale bar: 20 µm. **C, D** Western blotting for axin in hypotonic cell extracts. Red and blue arrow indicate the positions of phospho-variants and of the absent phospho-variant, respectively. **C** GFP-tagged proteins expressed in SW480 cells. Extracts were untreated (-) or CIP-treated. **D** SW480 cells expressing GFP-caxin^{RGS} were untreated (-) or treated with 0.4 µM okadaic acid (OA) for 6 hours. **A-C** Exchange of the DIX domain (caxin^{DIX}) decreased neither condensates nor phosphorylation, and serves as a negative control. All experiments were replicated at least three times.

Journal of Cell Science • Supplementary information

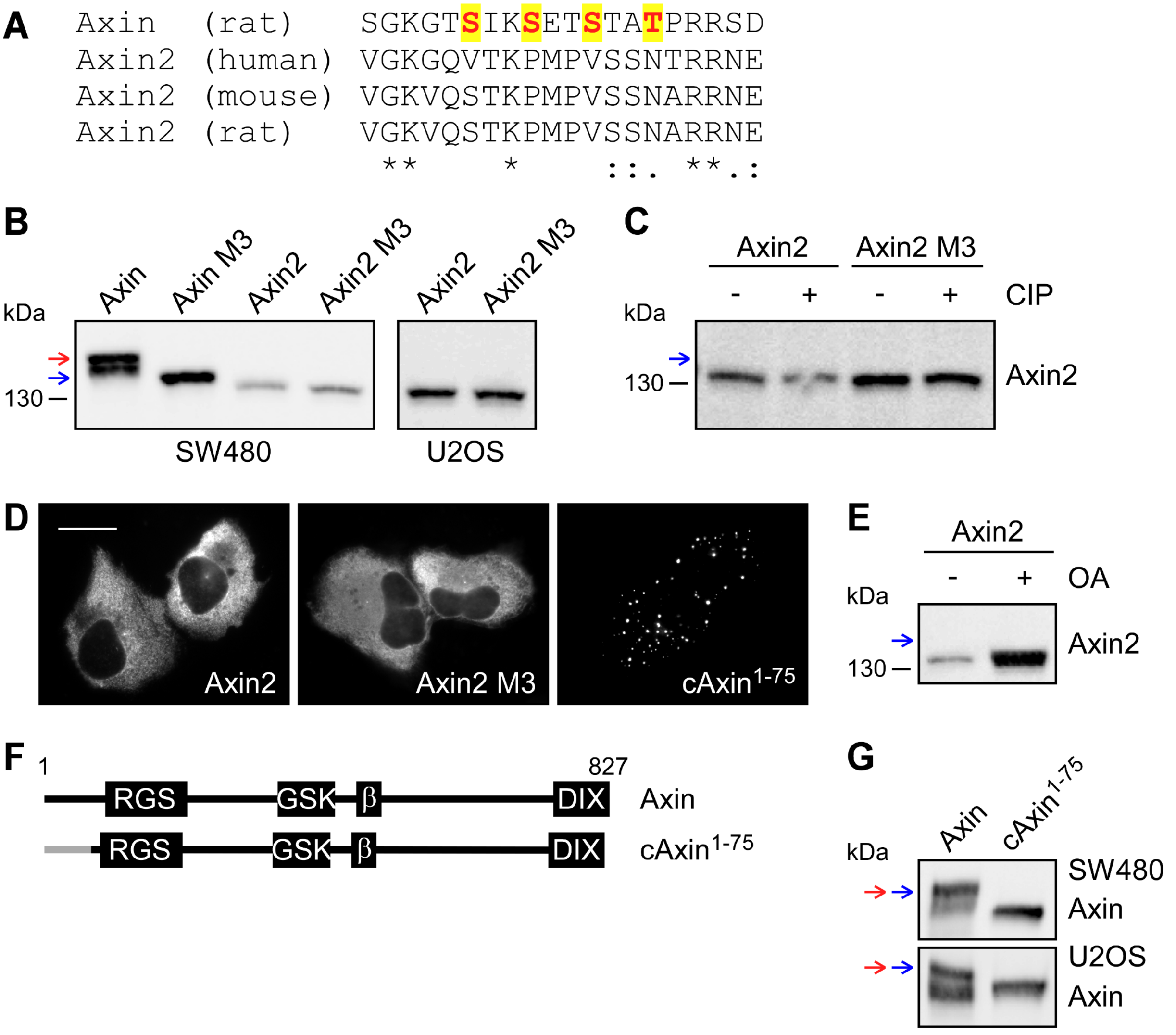

Fig. S4. CK1 phosphorylation is not conserved in axin2. A Clustal Omega alignment of axin (rat, UniProt identifier O70239) and axin2 (human Q9Y2T1, mouse O88566, rat O70240) sequences. Identity (*) and conservation between amino acid groups of strongly (:) and weakly (.) similar properties are indicated (Sievers et al., 2011). The Ser/Thr residues affecting the electrophoretic mobility of axin are highlighted. **B, C, E, G** Western blotting for axin2 and axin in hypotonic cell extracts. Red and blue arrow indicate the positions of phospho-variants and of the absent phospho-variant, respectively. **B** GFP-tagged proteins expressed in SW480 and U2OS cells. **C** GFP-tagged proteins expressed in SW480 cells. Extracts were untreated (-) or CIP-treated. **E** SW480 cells expressing GFP-axin2 were untreated (-) or treated with 0.4 µM okadaic acid (OA) for 6 hours. **G** SW480 and U2OS cells expressing indicated GFP-tagged proteins. **D** Fluorescence of indicated GFP-tagged proteins in U2OS cells. Scale bar: 20 µm. **F** To scale schemes of axin and the chimeric protein caxin$^{1-75}$. Parts of axin and axin2 are shown in black and grey, respectively. All experiments were replicated at least three times.

Journal of Cell Science • Supplementary information

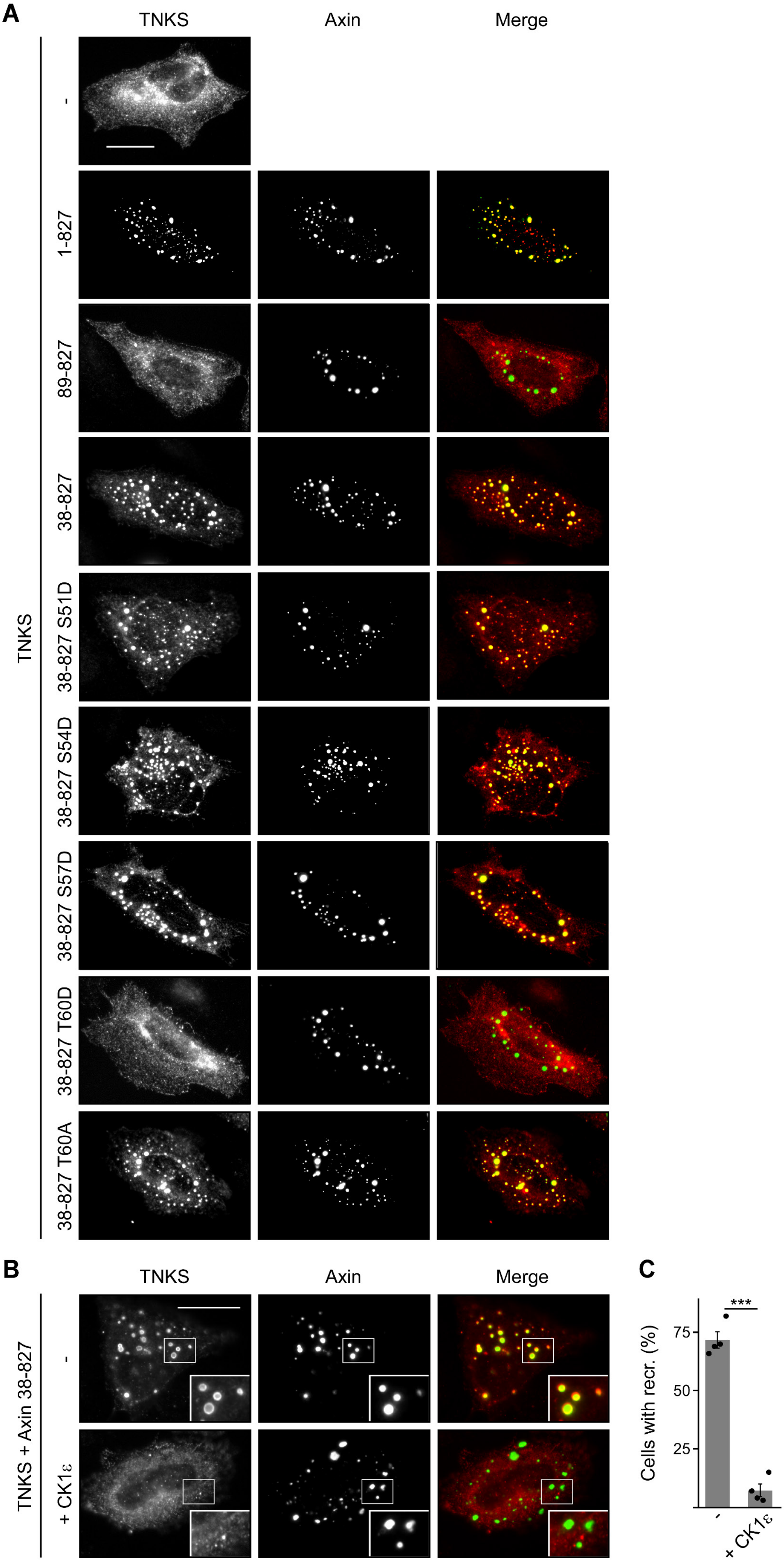

Journal of Cell Science • Supplementary information

Fig. S5. Phosphorylation at threonine 60 impaired tankyrase binding. A Immunofluorescence staining of Flag (red) and GFP fluorescence (green) in U2OS cells expressing Flag-tankyrase (TNKS) alone or together with the GFP-axin variants indicated on the left. Scale bar: 20 µm (A,B). The experiment was replicated four times. **B** Immunofluorescence staining of Flag (red) and GFP fluorescence (green) in U2OS cells expressing Flag-tankyrase (TNKS) and GFP-axin 38-827 without (-) or together with CK1ε. Insets are magnified at the lower right. **C** Percentage of cells with recruitment of tankyrase in axin condensates in the absence (-) or presence of CK1 out of 400 cells exhibiting axin condensates and tankyrase co-expression from four independent experiments, as shown in B (n=4). Results are mean +- s.e.m., *** $p<0.001$ (Student's *t*-test).

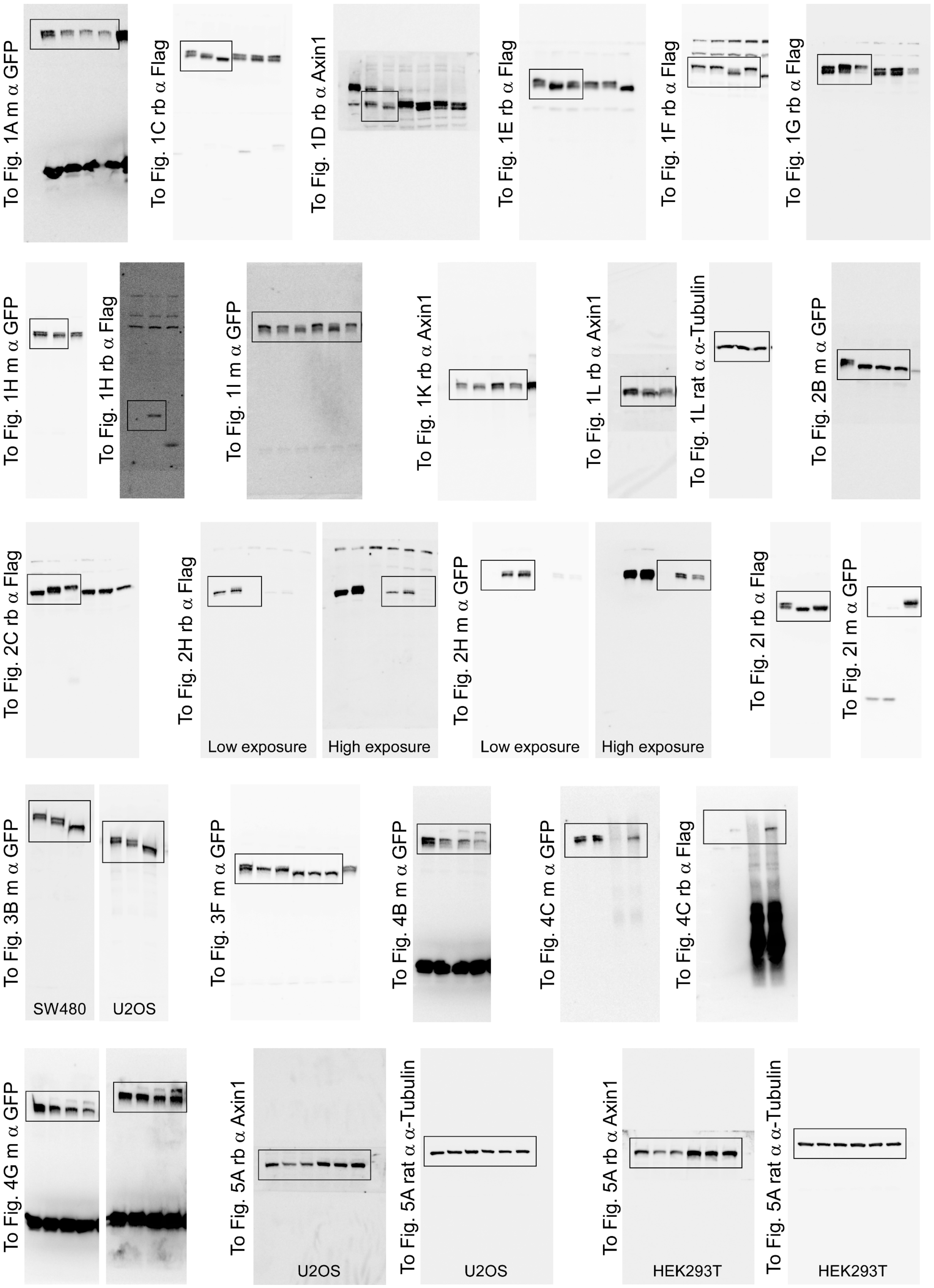

Fig. S6. Blot transparency. Uncropped blot images corresponding to the figures indicated on the left, supporting evaluation of antibody specificity (antibodies indicated on the left) and confirmation of band identification (boxes).
