# Supplementary material for: Phosphorylation of axin within biomolecular condensates counteracts its tankyrase-mediated degradation
Source: J Cell Sci. 2023 Oct 27;136(20):jcs261214. doi: 10.1242/jcs.261214 (PMC10652037; doi:10.1242/jcs.261214)
Supplement: Supplementary file 1 [file joces-136-261214_review_history.pdf]

Journal of Cell Science

Phosphorylation of axin within biomolecular condensates counteracts its tankyrase-mediated degradation

Katharina Klement, Martina Brückner and Dominic B. Bernkopf
DOI: 10.1242/jcs.261214

Editor: John Heath

Review timeline
Original submission: 31 March 2023
Editorial decision: 31 May 2023
First revision received: 18 August 2023
Accepted: 8 September 2023

Original submission

First decision letter

MS ID#: JOCES/2023/261214

MS TITLE: Axin phosphorylation in condensates counteracts tankyrase-mediated degradation

AUTHORS: Dominic B. Bernkopf
ARTICLE TYPE: Research Article

We have now reached a decision on the above manuscript.

To see the reviewers' reports and a copy of this decision letter, please go to: https://submit-jcs.biologists.org and click on the 'Manuscripts with Decisions' queue in the Author Area. (Corresponding author only has access to reviews.)

As you will see, the reviewers raise a number of substantial criticisms that prevent me from accepting the paper at this stage. They suggest, however, that a revised version might prove acceptable, if you can address their concerns. If you think that you can deal satisfactorily with the criticisms on revision, I would be pleased to see a revised manuscript. We would then return it to the reviewers.

Please ensure that you clearly highlight all changes made in the revised manuscript. Please avoid using 'Tracked changes' in Word files as these are lost in PDF conversion.

I should be grateful if you would also provide a point-by-point response detailing how you have dealt with the points raised by the reviewers in the 'Response to Reviewers' box. Please attend to all of the reviewers' comments. If you do not agree with any of their criticisms or suggestions please explain clearly why this is so.

Reviewer 1

Advance summary and potential significance to field

This study describes the molecular mechanism of Axin degradation in its condensate. The author identified the phosphorylation sites by CK1α in Axin and showed that its phosphorylation interfered with the binding of tankyrase resulting in the prevention of Axin degradation. Axin is the essential

© 2023. Published by The Company of Biologists under the terms of the Creative Commons Attribution License (https://creativecommons.org/licenses/by/4.0/).

molecule in the Wnt signaling pathway, and its stability directly affect the β-catenin stabilization. In this context, the results of this study would be important in the Wnt research field.

Comments for the author

The experiments are well designed and the results are clear and convincing.
There are several issues that the author should address to improve this study.

1. The separation of the upper and lower bands of Axin on SDS-PAGE is important in the experiment. The polyacrylamide gel concentration should be described.
2. Fig. 1C. By the treatment with CIP, the Axin molecular weight is smaller than that of the lower band, indicating that there are several phosphorylation sites in Axin. The author should comment on this point.
3. Fig. 1I. The phosphorylation of Axin by GSK3 has been reported as the author cited (Willert et al. 1999; Yamamoto et al. 1999). The author should test whether Axin is phosphorylated by GSK3 after pre-phosphorylated by CK1a?
4. Fig. 2D. There are at least seven CK1 family members. Did the author test whether CK1 members other than CK1a phosphorylate Axin?
5. Fig. 2I. Dvl2 expression reduced the phosphorylation of Axin. Under this condition, Axin is still condensated. The possible mechanism by which Axin phosphorylation is suppressed by Dvl2 in the Axin condensate should be discussed.
6. Wnt induces the recruitment of Axin complex to the LRP6 and b-catenin stabilization. Does Wnt stimulation induces Axin degradation? Does the time course of Axin phosphorylation and degradation in intact cells by Wnt stimulation should be shown.
7. Fig. 4C. The conclusion that phosphorylated Axin prevents the recruitment of tankyrase to Axin is important. When Axin was fully phosphorylated by the treatment with ocadaic acid or expression of CK1a? (Fig. 1G and J), is the interaction of Axin with tankyrase strongly suppressed?
8. Fig. 4G. The difference the interaction between Axin or AxinT60D and tankyrase should be shown quantitatively.

Reviewer 2

Advance summary and potential significance to field

This manuscript identifies an important phospho-regulatory site in Axin, a scaffold protein for the Wnt signaling pathway. Using cell culture assays, the author identified a phosphorylated Axin subpopulation that migrates distinctly on SDS-PAGE, and determined that this subpopulation was more stable than the dephosphorylated subpopulation. The author provided data suggesting that relevant kinase is CK1a and the phosphatase is PP1. In parallel, the author showed that disrupting Axin condensates shifts the population to the dephosphorylated state, suggesting that Axin condensation promotes phosphorylation. Immunofluorescence experiments indicate that CK1a colocalizes to Axin condensates and that PP1 is excluded, providing a possible explanation for why Axin condensation promotes phosphorylation. Finally, the author identified the precise Axin sequence motif phosphorylated by CK1a and determined that it overlaps with a tankyrase binding segment. The author concluded that phosphorylation of Axin by CK1a blocks tankyrase-mediated degradation.

Regulation of Axin stability is an important component of Wnt pathway regulation, and these results will be of broad interest to the cell signaling community. This manuscript should be published after the author addresses the comments below.

Comments for the author

Major Comments

1. A central claim of the paper is that PP1 is the phosphatase that dephosphorylates Axin (pg 5), but this claim needs more support. Two experiments support this claim. First, the phosphatase

© 2023. Published by The Company of Biologists under the terms of the Creative Commons Attribution License (https://creativecommons.org/licenses/by/4.0/).

inhibitor okadaic acid protected Axin from dephosphorylation. However, as noted by the author, okadaic acid can inhibit both PP1 and PP2A. Second, overexpression of PP1 decreased the level of phosphorylated Axin. However, this experiment does not conclusively exclude PP2A as the endogenous phosphatase because overexpressed phosphatases can dephosphorylate sites that may not be endogenous targets. It is important to further distinguish between PP1 and PP2A because a major point later in the paper is that PP1 is excluded from condensates, which explains how Axin phosphorylation is regulated. Further, there is a known PP2A binding site in Axin near the proposed CK1 phosphorylation site discussed in this manuscript (doi: 10.1073/pnas.2208787120). The author should consider the following additional experiments. Depending on the outcomes, one or both would be necessary to rigorously solidify the central claims of this manuscript. First the author could overexpress PP2A and assess whether it also dephosphorylates Axin. This experiment may require overexpressing multiple components of the PP2A holoenzyme. Second, the author could evaluate whether PP2A is excluded from condensates, because if both PP1 and PP2A are excluded then it is less critical to distinguish which is the endogenous phosphatase.

2. Another central point of the paper is that CK1a is the kinase that phosphorylates Axin. To establish this point, the author uses small-molecule inhibitors D4476 and IC261 to inhibit CK1a (Fig 1I). Both inhibitors target multiple CK1 isoforms, and IC261 preferentially targets the delta and epsilon forms of CK1 (doi: 10.1074/jbc.M001713200). CK1e has been reported to interact with Axin (see for example doi: 10.1074/jbc.M105148200), so it is important to distinguish between CK1 isoforms. Later in the manuscript, the key experiment showing that CK1 is localizes to Axin condensates was performed with CK1a (Fig 2F). Because other CK1 isoforms are known to interact with Axin, it is plausible that they would also localize to Axin condensates. New experiments may not be necessary, but potential roles for other CK1 isoforms should be explicitly considered and discussed.

3. On pg 7, the author writes: “Interestingly, the upper axin band also disappeared upon co-expression of the positive Wnt pathway regulator Dvl2 indicative for decreased phosphorylation (Fig. 2I). As incorporation of Dvl2 in axin condensates (Fig. 2J) increases condensates' dynamics (Schwarz-Romond et al., 2007), this change in axin condensates' properties may suffice to impair axin phosphorylation.” It is unclear what exactly is meant by “dynamics” and how that would affect phosphorylation. On pg 12, the manuscript elaborates: “Dvl2 integrates in axin condensates and increases their dynamics (Schwarz-Romond et al., 2007), meaning that axin molecules shuttle more frequently on and off the condensates, which increases the residence time of axin molecules outside condensates, where they become more accessible for PP1.” The 2007 reference measured fluorescence recovery after photobleaching, which provides a measure of exchange rates. These measurements do not directly report on the steady-state distribution of Axin inside or outside condensates, and it is not necessarily true that changing dynamics will increase the residence time of Axin outside of condensates. It is therefore problematic to use this logic to suggest that changes in condensate dynamics affect Axin phosphorylation.

Minor comments

1. The manuscript is written in an unusually casual narrative style that sometimes hinders a clear understanding of the logic. For example, on page 5 the author writes: “I got interested to explore the nature of these axin variants”. It is unclear what this statement means. The underlying points can be followed but the manuscript would benefit from removing vague, subjective statements and replacing them with clear logical connections.

2. The first sentence of the results section is difficult to follow. On pg 5, the author writes: “When I analyzed degradation of transiently expressed axin, I noticed….” It takes several lines to find a description of the cycloheximide inhibition experiment, and even that statement is missing key details to orient the reader. The manuscript would be easier to follow if it opened with a clear statement of the observation and provided some context for what type of experiment is being conducted. Here is a possible alternative: “In cell culture assays with transiently expressed Axin, I observed a distinct double band for Axin on Western blots. The Axin variant in the upper band exhibited a markedly increased band intensity compared to the variant in the lower band upon inhibition of translation by cycloheximide (Fig. 1A). This observation suggests that the upper band variant is degraded more slowly than the lower band variant.”

3. Gel images in all figures are narrowly cropped. Many journals are now requesting that full, uncropped blot images be provided in supplemental figures at minimum for a representative subset

© 2023. Published by The Company of Biologists under the terms of the Creative Commons Attribution License (https://creativecommons.org/licenses/by/4.0/).

of figures. This practice helps readers to evaluate antibody specificity and confirm band identification at the appropriate molecular weight.

4. In Fig 1C & D, calf intestinal phosphatase (CIP) treatment appears to produce a third, faster-migrating species of Axin. Presumably this band is a fully dephosphorylated state where CIP has removed additional phosphatases elsewhere on Axin. This point should be explicitly acknowledged in the text (pg 5) with citations to literature reports of other known Axin phosphosites. This point is also relevant for the Axin M3 mutant (Fig 2 & pg 6).

5. In Fig 1I, can the author elaborate on the choice of inhibitor concentrations? Specifically, why was a single 5 μM concentration chosen for BIO while the other inhibitors were tested at both 10 and 100 μM? Is this choice based on known affinities for these inhibitors?

6. On pg 11, the author writes: "Moreover, a partial rescue of condensate formation by recruiting axin M3 in WT axin condensates partially rescued phosphorylation as well (Fig. 2G, H)." Fig. 2G shows fluorescence microscopy data with GFP tags on both wild-type Axin and Axin-M3. This data cannot be used to conclude that Axin-M3 is recruited to condensates. If establishing this point is critical, orthogonal fluorescence tags could be used.

First revision

Author response to reviewers' comments

As any formatting of the text is lost here, the point-by-point response will also be uploaded as a PDF file.

Point-by-Point response (green) to the reviewers comments (black)

Reviewer 1 Advance Summary and Potential Significance to Field:
This study describes the molecular mechanism of Axin degradation in its condensate. The author identified the phosphorylation sites by CK1α in Axin and showed that its phosphorylation interfered with the binding of tankyrase, resulting in the prevention of Axin degradation. Axin is the essential molecule in the Wnt signaling pathway, and its stability directly affect the β-catenin stabilization. In this context, the results of this study would be important in the Wnt research field.

Reviewer 1 Comments for the Author:
The experiments are well designed and the results are clear and convincing.
There are several issues that the author should address to improve this study.

We thank the reviewer for her/his interest in our work and the constructive suggestions to improve our study.

General comment: The numbering of pages and figures may have changed compared to the first manuscript version due to requested changes in formatting and due to new experimental data. The page numbers and figure numbers in the point-by-point response below refer to the new manuscript.

1. The separation of the upper and lower bands of Axin on SDS-PAGE is important in the experiment. The polyacrylamide gel concentration should be described.

We added a sentence describing the polyacrylamide gel concentration (8%) to the Western blot paragraph of the Materials and Methods section.

2. Fig. 1C. By the treatment with CIP, the Axin molecular weight is smaller than that of the lower band, indicating that there are several phosphorylation sites in Axin. The author should comment on this point.

© 2023. Published by The Company of Biologists under the terms of the Creative Commons Attribution License (https://creativecommons.org/licenses/by/4.0/).

Yes, we fully agree that the molecular weight of CIP-treated axin is smaller than that of the lower band, and that this indicates additional phosphorylation sites. This is in line with literature reports describing several phosphorylation sites in axin. For clarification, we added a respective sentence on page 4 with citations of the literature reports.

3. Fig. 1I. The phosphorylation of Axin by GSK3 has been reported as the author cited (Willert et al. 1999; Yamamoto et al. 1999). The author should test whether Axin is phosphorylated by GSK3 after pre-phosphorylated by CK1a?

We agree that it would be generally interesting to investigate whether axin is phosphorylated via a dual-kinase mechanism similarly as β-catenin (Liu et al., 2002), if this was the intention of the reviewer. However, this is not a central point of our study, since the investigated phospho-axin variant was independent from GSK3 (Figs. 1I, S1B). Moreover, in vitro phosphorylation of purified proteins would have to be performed to address the time sequence of the phosphorylation events, which is out of scope of the study. For these two reasons, we abstained from investigating consecutive GSK3 phosphorylation.
Besides, reported data already imply that phosphorylation of axin by CK1 at different sites affects phosphorylation by GSK3 (Luo et al., 2007).

4. Fig. 2D. There are at least seven CK1 family members. Did the author test whether CK1 members other than CK1a phosphorylate Axin?

Upon overexpression, also CK1ε phosphorylated axin (new Fig. S1C), showing that also other CK1 members can phosphorylate axin in principal. However, newly performed knockdown experiments revealed that CK1α is indeed the most relevant endogenous kinase, as knockdown of CK1α was sufficient to induce dephosphorylation of axin while knockdown of CK1δ or CK1ε had no effect (new Figs. 1K, S1D).

5. Fig. 2I. Dvl2 expression reduced the phosphorylation of Axin. Under this condition, Axin is still condensated. The possible mechanism by which Axin phosphorylation is suppressed by Dvl2 in the Axin condensate should be discussed.

Since dephosphorylation of axin by Dvl2 is an interesting finding, we elaborated on the possible mechanism. As shown before, Dvl2 efficiently incorporates in axin condensates (Fig. S2C,E). Notably, incorporation of Dvl2 in axin condensates promoted recruitment of PP1 (new Fig. 2J,K), potentially explaining how Dvl2 induced dephosphorylation of axin without dissolving its condensates. PP1 was also recruited in homotypic condensates formed by Dvl2 alone (Fig. S2D,F). Thus, Dvl2 may promote recruitment of PP1 into axin condensates by interacting with both proteins. Alternatively, incorporation of Dvl2 in axin condensates may alter their selectivity by changing condensate properties, such as dynamics.

6. Wnt induces the recruitment of Axin complex to the LRP6 and b-catenin stabilization. Does Wnt stimulation induces Axin degradation? Does the time course of Axin phosphorylation and degradation in intact cells by Wnt stimulation should be shown.

We appreciate the interest of the reviewer to extent our study towards Wnt-stimulated axin degradation. Yes, it is already known that Wnt stimulation induces axin degradation (Ji et al., 2017; Li et al., 2012; Willert et al., 1999; Yamamoto et al., 1999) and Wnt-induced degradation of axin is associated with dephosphorylation of axin (Ji et al., 2017; Willert et al., 1999; Yamamoto et al., 1999). Our proposed new mechanism may well contribute to Wnt- induced axin degradation, either generically or under specific circumstances. However, since Wnt signaling promotes dephosphorylation of axin at several sites (Ji et al., 2017; Kim et al., 2013; Willert et al., 1999; Yamamoto et al., 1999), linking of any phenotype specifically to our new mechanism would require intensive studies. As this is out of scope for our study and as Wnt-induced degradation and preceding dephosphorylation of axin are already published, we prefer not to include new data addressing this issue.

7. Fig. 4C. The conclusion that phosphorylated Axin prevents the recruitment of tankyrase to Axin is important. When Axin was fully phosphorylated by the treatment with ocadaic acid or expression

© 2023. Published by The Company of Biologists under the terms of the Creative Commons Attribution License (https://creativecommons.org/licenses/by/4.0/).

of CK1a? (Fig. 1G and J), is the interaction of Axin with tankyrase strongly suppressed?

Ocadaic acid treatment or expression of CK1 did not convincingly reduce binding of full length axin in immunoprecipitation assays. Here, differences in interaction between the phospho-variants only became apparent, when non-phosphorylated axin and phosphorylated axin competed for tankyrase binding in the same sample (Fig 4C).

As described (p. 8), axin contains two segments for tankyrase binding (A aa 18-30 and B aa 60-79). Our data suggest that phosphorylation of T60 reduces binding of segment B, which overlaps with the phosphorylation site. Thus, constitutive binding via segment A may hide phosphorylation-dependent differences in binding of full length axin.

To address the question of whether full phosphorylation of axin by CK1 expression strongly suppresses interaction with tankyrase, we performed co-localization experiments with tankyrase and axin 38-827. As segment A is deleted from axin 38-827, this experiment allowed to investigate specifically binding of the axin segment B to tankyrase (Figs. 4D,E, S5A). Indeed, co-expression of CK1 almost completely abolished the recruitment of tankyrase into axin 38-827 condensates, suggesting that phosphorylation strongly suppressed tankyrase binding (new Fig. S5B,C).

Of note, our combined data strongly suggest that reduced binding of phosphorylated segment B to tankyrase is of functional importance for tankyrase-mediated degradation of axin (Figs. 1A,L, 4B,G,H, 5A,B), irrespective of binding via segment A. Potentially, simultaneous binding via both segments is required for proper degradation.

8. Fig. 4G. The difference the interaction between Axin or AxinT60D and tankyrase should be shown quantitatively.

A quantification of Fig. 4G is shown in Fig. 4H.

Ji, L., B. Jiang, X. Jiang, O. Charlat, A. Chen, C. Mickanin, A. Bauer, W. Xu, X. Yan, and F. Cong. 2017. The SIAH E3 ubiquitin ligases promote Wnt/beta-catenin signaling through mediating Wnt- induced Axin degradation. *Genes Dev*. 31:904-915.
Kim, S.E., H. Huang, M. Zhao, X. Zhang, A. Zhang, M.V. Semonov, B.T. MacDonald, X. Zhang, J. Garcia Abreu, L. Peng, and X. He. 2013. Wnt stabilization of beta-catenin reveals principles for morphogen receptor-scaffold assemblies. *Science*. 340:867-870.
Li, V.S., S.S. Ng, P.J. Boersema, T.Y. Low, W.R. Karthaus, J.P. Gerlach, S. Mohammed, A.J. Heck, M.M. Maurice, T. Mahmoudi, and H. Clevers. 2012. Wnt signaling through inhibition of beta- catenin degradation in an intact Axin1 complex. *Cell*. 149:1245-1256.
Liu, C., Y. Li, M. Semenov, C. Han, G.H. Baeg, Y. Tan, Z. Zhang, X. Lin, and X. He. 2002. Control of beta- catenin phosphorylation/degradation by a dual-kinase mechanism. *Cell*. 108:837-847.
Luo, W., A. Peterson, B.A. Garcia, G. Coombs, B. Kofahl, R. Heinrich, J. Shabanowitz, D.F. Hunt, H.J. Yost, and D.M. Virshup. 2007. Protein phosphatase 1 regulates assembly and function of the beta-catenin degradation complex. *EMBO J*. 26:1511-1521.
Willert, K., S. Shibamoto, and R. Nusse. 1999. Wnt-induced dephosphorylation of axin releases beta-catenin from the axin complex. *Genes Dev*. 13:1768-1773.
Yamamoto, H., S. Kishida, M. Kishida, S. Ikeda, S. Takada, and A. Kikuchi. 1999. Phosphorylation of axin, a Wnt signal negative regulator, by glycogen synthase kinase-3beta regulates its stability. *J Biol Chem*. 274:10681-10684.

Reviewer 2 Advance Summary and Potential Significance to Field:
This manuscript identifies an important phospho-regulatory site in Axin, a scaffold protein for the Wnt signaling pathway. Using cell culture assays, the author identified a phosphorylated Axin subpopulation that migrates distinctly on SDS-PAGE, and determined that this subpopulation was more stable than the dephosphorylated subpopulation. The author provided data suggesting that relevant kinase is CK1a and the phosphatase is PP1. In parallel, the author showed that disrupting Axin condensates shifts the population to the dephosphorylated state, suggesting that Axin condensation promotes phosphorylation. Immunofluorescence experiments indicate that CK1a colocalizes to Axin condensates and that PP1 is excluded, providing a possible explanation for why Axin condensation promotes phosphorylation. Finally, the author identified the precise Axin

© 2023. Published by The Company of Biologists under the terms of the Creative Commons Attribution License (https://creativecommons.org/licenses/by/4.0/).

sequence motif phosphorylated by CK1a and determined that it overlaps with a tankyrase binding segment. The author concluded that phosphorylation of Axin by CK1a blocks tankyrase-mediated degradation.

Regulation of Axin stability is an important component of Wnt pathway regulation, and these results will be of broad interest to the cell signaling community. This manuscript should be published after the author addresses the comments below.

We thank the reviewer for her/his detailed thoughts on our study and the constructive suggestions for further improvements.

General comment: The numbering of pages and figures may have changed compared to the first manuscript version due to requested changes in formatting and due to new experimental data. The page numbers and figure numbers in the point-by-point response below refer to the new manuscript.

Reviewer 2 Comments for the Author:
Major Comments

1. A central claim of the paper is that PP1 is the phosphatase that dephosphorylates Axin (pg 5), but this claim needs more support. Two experiments support this claim. First, the phosphatase inhibitor okadaic acid protected Axin from dephosphorylation. However, as noted by the author, okadaic acid can inhibit both PP1 and PP2A. Second, overexpression of PP1 decreased the level of phosphorylated Axin. However, this experiment does not conclusively exclude PP2A as the endogenous phosphatase because overexpressed phosphatases can dephosphorylate sites that may not be endogenous targets. It is important to further distinguish between PP1 and PP2A because a major point later in the paper is that PP1 is excluded from condensates, which explains how Axin phosphorylation is regulated. Further, there is a known PP2A binding site in Axin near the proposed CK1 phosphorylation site discussed in this manuscript (doi: 10.1073/pnas.2208787120). The author should consider the following additional experiments. Depending on the outcomes, one or both would be necessary to rigorously solidify the central claims of this manuscript. First, the author could overexpress PP2A and assess whether it also dephosphorylates Axin. This experiment may require overexpressing multiple components of the PP2A holoenzyme. Second, the author could evaluate whether PP2A is excluded from condensates, because if both PP1 and PP2A are excluded then it is less critical to distinguish which is the endogenous phosphatase.

We obtained expression plasmids for the catalytic PP2A subunit (PP2A-C) and the two regulatory subunits PP2A-Aα and PP2A-Aβ and cloned them into the Flag-tag expression vector for direct comparison with Flag-CK1α (positive control for recruitment) and Flag-PP1 (negative control for recruitment). PP2A-C, PP2A-Aα and PP2A-Aβ were excluded from axin condensates. Similarly, combinations of PP2A-C expressed together with either PP2A-Aα or PP2A-Aβ were excluded from axin condensates (new Fig. S2A,B), suggesting that both PP2A and PP1 are excluded from axin condensates.

2. Another central point of the paper is that CK1a is the kinase that phosphorylates Axin. To establish this point, the author uses small-molecule inhibitors D4476 and IC261 to inhibit CK1a (Fig 1I). Both inhibitors target multiple CK1 isoforms, and IC261 preferentially targets the delta and epsilon forms of CK1 (doi: 10.1074/jbc.M001713200). CK1e has been reported to interact with Axin (see for example doi: 10.1074/jbc.M105148200), so it is important to distinguish between CK1 isoforms. Later in the manuscript, the key experiment showing that CK1 is localizes to Axin condensates was performed with CK1a (Fig 2F). Because other CK1 isoforms are known to interact with Axin, it is plausible that they would also localize to Axin condensates. New experiments may not be necessary, but potential roles for other CK1 isoforms should be explicitly considered and discussed.

To address this issue we performed additional overexpression and knockdown experiments to investigate other CK1 isoforms. Upon overexpression, CK1ε phosphorylated axin similarly as CK1α (new Fig. S1C), consistent with the published axin-CK1ε interaction cited by the reviewer.

© 2023. Published by The Company of Biologists under the terms of the Creative Commons Attribution License (https://creativecommons.org/licenses/by/4.0/).

However, knockdown experiments revealed that CK1α is the most relevant endogenous isoform, as knockdown of CK1α was sufficient to induce dephosphorylation of axin while knockdown of CK1δ or CK1ε had now effect (new Figs. 1K, S1D).

3. On pg 7, the author writes: “Interestingly, the upper axin band also disappeared upon co-expression of the positive Wnt pathway regulator Dvl2 indicative for decreased phosphorylation (Fig. 2I). As incorporation of Dvl2 in axin condensates (Fig. 2J) increases condensates' dynamics (Schwarz-Romond et al., 2007), this change in axin condensates' properties may suffice to impair axin phosphorylation.” It is unclear what exactly is meant by “dynamics” and how that would affect phosphorylation. On pg 12, the manuscript elaborates: “Dvl2 integrates in axin condensates and increases their dynamics (Schwarz- Romond et al., 2007), meaning that axin molecules shuttle more frequently on and off the condensates, which increases the residence time of axin molecules outside condensates, where they become more accessible for PP1.” The 2007 reference measured fluorescence recovery after photobleaching, which provides a measure of exchange rates. These measurements do not directly report on the steady-state distribution of Axin inside or outside condensates, and it is not necessarily true that changing dynamics will increase the residence time of Axin outside of condensates. It is therefore problematic to use this logic to suggest that changes in condensate dynamics affect Axin phosphorylation.

We agree with the reviewer that the measurement of exchange rates by FRAP does not directly report on the steady-state distribution of axin, and we elaborated on the potential Dvl2 function. As shown before, Dvl2 efficiently incorporates in axin condensates (Fig. S2C,E). Notably, incorporation of Dvl2 in axin condensates promoted recruitment of PP1 (new Fig. 2J,K), potentially explaining how Dvl2 induced dephosphorylation of axin without dissolving its condensates. PP1 was also recruited in homotypic condensates formed by Dvl2 alone (Fig. S2D,F). Thus, Dvl2 may promote recruitment of PP1 into axin condensates by interacting with both proteins. Alternatively, incorporation of Dvl2 in axin condensates may alter their selectivity by changing condensate properties, such as condensate dynamics.

Minor comments

1. The manuscript is written in an unusually casual narrative style that sometimes hinders a clear understanding of the logic. For example, on page 5 the author writes: “I got interested to explore the nature of these axin variants”. It is unclear what this statement means. The underlying points can be followed, but the manuscript would benefit from removing vague, subjective statements and replacing them with clear logical connections.

We thank the reviewer for this consideration. To address the issue, subjective or narrative statements were deleted or replaced by more scientific terms. In detail:
“I got interested to explore the nature of these axin variants” was deleted (page 4); “Giving a first hint” was replaced by “providing first evidence” (page 4);
“Thus, I concluded” was replaced by “These findings suggested” (page 5); “I wanted to investigate” was replaced by “we investigated” (page 5); “My observations with” was deleted (page 7);
“I speculated that” was deleted (page 7);
“Which I noticed at the beginning of the study” was deleted (page 8).

2. The first sentence of the results section is difficult to follow. On pg 5, the author writes: “When I analyzed degradation of transiently expressed axin, I noticed….” It takes several lines to find a description of the cycloheximide inhibition experiment, and even that statement is missing key details to orient the reader. The manuscript would be easier to follow if it opened with a clear statement of the observation and provided some context for what type of experiment is being conducted. Here is a possible alternative: “In cell culture assays with transiently expressed Axin, I observed a distinct double band for Axin on Western blots. The Axin variant in the upper band exhibited a markedly increased band intensity compared to the variant in the lower band upon inhibition of translation by cycloheximide (Fig. 1A). This observation suggests that the upper band variant is degraded more slowly than the lower band variant.”

We made respective changes following the provided alternative.

3. Gel images in all figures are narrowly cropped. Many journals are now requesting that full,

© 2023. Published by The Company of Biologists under the terms of the Creative Commons Attribution License (https://creativecommons.org/licenses/by/4.0/).

uncropped blot images be provided in supplemental figures, at minimum for a representative subset of figures. This practice helps readers to evaluate antibody specificity and confirm band identification at the appropriate molecular weight.

We provided uncropped blot images in the new supplemental figure S6, as requested.

4. In Fig 1C & D, calf intestinal phosphatase (CIP) treatment appears to produce a third, faster-migrating species of Axin. Presumably this band is a fully dephosphorylated state where CIP has removed additional phosphatases elsewhere on Axin. This point should be explicitly acknowledged in the text (pg 5) with citations to literature reports of other known Axin phosphosites. This point is also relevant for the Axin M3 mutant (Fig 2 & pg 6).

We added a respective explanatory sentence with citations to literature reports, as suggested (see page 4).

5. In Fig 1I, can the author elaborate on the choice of inhibitor concentrations? Specifically, why was a single 5 µM concentration chosen for BIO while the other inhibitors were tested at both 10 and 100 µM? Is this choice based on known affinities for these inhibitors?

As we are using BIO more frequently in our lab than D4476 or IC261, we knew that 5 µM BIO inhibits GSK3 efficiently and we decided to test a certain concentration range for the other inhibitors to ensure efficient inhibition of CK1. Indeed, the choice of higher concentrations for D4476 and IC261 compared to BIO is based on known affinities for the inhibitors: IC50: BIO 5 nM, D4476 200-300 nM, IC261 1600 nM, according to www.selleckchem.com.

6. On pg 11, the author writes: "Moreover, a partial rescue of condensate formation by recruiting axin M3 in WT axin condensates partially rescued phosphorylation as well (Fig. 2G, H)." Fig. 2G shows fluorescence microscopy data with GFP tags on both wild-type Axin and Axin-M3. This data cannot be used to conclude that Axin-M3 is recruited to condensates. If establishing this point is critical, orthogonal fluorescence tags could be used.

This is a misunderstanding: in Fig. 2G, Axin M3 is tagged with Flag and WT Axin is tagged with GFP, as indicated on the left. All images show immunofluorescence staining of the Flag-tag visualizing Axin M3 only, as indicated on top. Thus, the condensates in the lower row are only visualized by Axin M3 that was recruited into WT Axin condensates, as Axin M3 was not capable to form condensates on itself (upper row). The GFP fluorescence of GFP-Axin WT, which was co-expressed in the lower row, is not shown, as its presence becomes apparent by the appearance of the typical condensate pattern. We now stated this explicitly in the figure legend, to avoid a similar misunderstanding by future readers.

Second decision letter

MS ID#: JOCES/2023/261214

MS TITLE: Axin phosphorylation in condensates counteracts tankyrase-mediated degradation

AUTHORS: Katharina Klement, Martina Brueckner, and Dominic B. Bernkopf
ARTICLE TYPE: Research Article

I am happy to tell you that your manuscript has been accepted for publication in Journal of Cell Science, pending standard ethics checks.

© 2023. Published by The Company of Biologists under the terms of the Creative Commons Attribution License (https://creativecommons.org/licenses/by/4.0/).

Reviewer 1

Advance summary and potential significance to field

This study describes the molecular mechanism of Axin degradation in its condensate. The author identified the phosphorylation sites by CK1alpha in Axin and showed that its phosphorylation interfered with the binding of tankyrase resulting in the prevention of Axin degradation. Axin is the essential molecule in the Wnt signaling pathway, and its stability directly affect the beta-catenin stabilization. In this context, the results of this study would be important in the Wnt research field.

Comments for the author

According to my critiques, the authors improved the manuscript. I have no further comments.

Reviewer 2

Advance summary and potential significance to field

The authors have satisfactorily addressed most of the reviewer comments. However the authors should address one major outstanding issue before publication. The authors will need to evaluate if further new experiments are necessary.

Comments for the author

Major Comments

In their initial manuscript, the authors demonstrated that the phosphatase PP1 was excluded from Axin condensates. In the revised manuscript, the authors performed experiments to evaluate if PP2A is also excluded from Axin condensates. This experiment is important because PP2A could be the phosphatase that dephosphorylates Axin in vivo. However, there is a major problem in the authors' new experiment as presented. The authors overexpressed the PP2A-C subunit (catalytic domain) and two different isoforms of an A subunit (PP2A-α and PP2A-Aβ). PP2A is a holoenzyme consisting of A, B, and C subunits. The authors did not deliver a B subunit, and the constructs they delivered should not form a functional holoenzyme with an A, B, and C subunit. This point is critical because the B subunit (specifically B56) is reported to interact with Axin (1,2). The correct experiment would be to overexpress PP2A-Aα,* B56, and PP2A-C.

At minimum, the authors must clearly explain their reasoning for delivering two A subunit isoforms and omitting the critical B subunit.

The authors could make the case that the PP2A-C overexpression experiment alone is sufficient to evaluate if PP2A-C localizes to Axin condensates. However, this claim would be difficult to justify because it is unclear if overexpressed PP2A-C would interact with Axin condensates in the absence of roughly stoichiometric B56 subunit.

*PP2A-Aα has been reported to assemble a holoenzyme complex with B56 in structural studies (3,4).

References

(1) Cantoria, M. J.; Alizadeh, E.; Ravi, J.; Varghese, R. P.; Bunnag, N.; Pond K. W.; Kettenbach, A. N.; Ahmed, Y.; Paek, A. L.; Tyson, J. J.; Doubrovinski K.; Lee, E.; Thorne, C. A. Feedback in the β-Catenin Destruction Complex Imparts Bistability and Cellular Memory. Proc National Acad Sci 2023, 120 (2), e2208787120. https://doi.org/10.1073/pnas.2208787120.
(2) Hertz, E. P. T.; Kruse, T.; Davey, N. E.; Mendez, B. L.; Sigurðsson, J. O.; Montoya, G.; Olsen, J. V.; Nilsson, J. A Conserved Motif Provides Binding Specificity to the PP2A-B56 Phosphatase. Mol. Cell 2016, 63 (4), 686–695. https://doi.org/10.1016/j.molcel.2016.06.024.

© 2023. Published by The Company of Biologists under the terms of the Creative Commons Attribution License (https://creativecommons.org/licenses/by/4.0/).

(3) Leonard, D.; Huang, W.; Izadmehr, S.; O'Connor, C. M.; Wiredja, D. D.; Wang Z.; Zaware, N.; Chen, Y.; Schlatzer, D. M.; Kiselar, J.; Vasireddi, N.; Schüchner, S.; Perl, A. L.; Galsky, M. D.; Xu, W.; Brautigan, D. L.; Ogris, E.; Taylor, D. J.; Narla, G. Selective PP2A Enhancement through Biased Heterotrimer Stabilization. Cell 2020, 181 (3), 688-701.e16. https://doi.org/10.1016/j.cell.2020.03.038.
(4) Cho, U.-S.; Xu, W. Crystal Structure of a Protein Phosphatase 2A Heterotrimeric Holoenzyme. Nature 2007, 445 (7123), 53–57. https://doi.org/10.1038/nature05351.

© 2023. Published by The Company of Biologists under the terms of the Creative Commons Attribution License (https://creativecommons.org/licenses/by/4.0/).
